# Inward- versus outward-focused bioeconomy strategies for British Columbia’s forest products industry: a harvested wood products carbon storage and emission perspective

**DOI:** 10.1186/s13021-021-00193-4

**Published:** 2021-09-25

**Authors:** Sheng H. Xie, Werner A. Kurz, Paul N. McFarlane

**Affiliations:** 1 Pacific Institute for Climate Solutions, University House 1, 2489 Sinclair Rd, Victoria, BC V8N 6M2 Canada; 2 Faculty of Forestry, University of British Columbia, 2424 Main Mall, Vancouver, BC V6T 1Z4 Canada; 3 Natural Resources Canada, Canadian Forest Service, 506 Burnside Road West, Victoria, BC V8Z 1M5 Canada

**Keywords:** Climate change mitigation, Emission reduction, Carbon dynamics modeling, Harvested wood products, Bioeconomy, Mass timber construction, Biofuel, Pulp and paper, Wood pellets

## Abstract

**Background:**

British Columbia’s (BC) extensive forest resources provide climate change mitigation opportunities that are available to few other jurisdictions. However, as a consequence of the Mountain Pine Beetle outbreak and large-scale wildfires, BC is anticipating reduced roundwood harvest for the next decades. Progress towards more climatically efficient utilization of forest resources is needed. This research quantitatively compared the greenhouse gas emission consequences of nine harvested wood products trade and consumption strategies. Inward-focused strategies use wood products within Canada to achieve emission reduction objectives, while outward-focused strategies encourage exports of wood products.

**Results:**

In the business-as-usual baseline scenario, average emissions arising from BC-originated harvested wood products between 2016 and 2050 were 40 MtCO_2_e yr^−1^. The estimated theoretical boundaries were 11 MtCO_2_e yr^−1^ and 54 MtCO_2_e yr^−1^, under the scenarios of using all harvests for either construction purposes or biofuel production, respectively. Due to the constrained domestic market size, inward-focused scenarios that were based on population and market capacity achieved 0.3–10% emission reductions compared to the baseline. The international markets were larger, however the emissions varied substantially between 68% reduction and 25% increase depending on wood products’ end uses.

**Conclusions:**

Future bioeconomy strategies can have a substantial impact on emissions. This analysis revealed that from a carbon storage and emission perspective, it was better to consume BC’s harvests within Canada and only export those products that would be used for long-lived construction applications, provided that construction market access beyond the US was available. However, restricting export of wood products destined for short-lived uses such as pulp and wood pellets would have significant economic and social impacts. On the other hand, inward-focused strategies had a small but politically and environmentally meaningful contribution to BC’s climate action plan. This study also revealed the conflicts between a demand-driven bioeconomy and targeted environmental outcomes. A hierarchical incentive system that could co-exist with other market drivers may help achieve emission reduction goals, but this would require a better quantitative understanding of wood products’ substitution effects. While the analyses were conducted for BC, other regions that are net exporters of wood products may face similar issues.

**Supplementary Information:**

The online version contains supplementary material available at 10.1186/s13021-021-00193-4.

## Background

Much of the world is seeking to combat the threat of climate change and to achieve Sustainable Development Goals (SDGs), such as ensuring access to sustainable modern energy and building sustainable cities and communities [1, 2]. Different jurisdictions possess widely divergent levels of resources and may have to find different solutions to sustainable energy and low impact construction techniques.

The province of British Columbia (BC) in Canada is well endowed with one of the largest forest areas per capita in the world: 11.2 ha per person [3, 4]. These forest resources provide climate change mitigation opportunities that many other jurisdictions do not have and they are expected to contribute to meeting the province’s and Canada’s emission reduction targets.

Forests contribute to emission reduction by removing and storing atmospheric carbon [5, 6]. Humans harvest forest biomass, process it into harvested wood products (HWPs), and use wood for energy. Carbon is locked up in HWPs until they are fully or partially decomposed or combusted. Simultaneously, new trees continue to grow and sequester carbon. Depending on the products’ characteristics and their end uses, the carbon pools in HWPs may effectively delay the release of carbon to the atmosphere [7]. The size of the HWP carbon pool fluctuates depending on the service times of various end uses as well as society’s consumption capacity and disposition practices of HWPs [8–10].

The majority of the carbon harvested from BC’s forests is stored in HWPs for a certain period^1^ while 38% is used to provide energy and 1% is unrecoverable material loss [11–15]. However, with time, much of the carbon stored in HWPs will eventually be emitted back to the atmosphere, with the exception of some extreme cases such as wooden temples that have stood for over a thousand years (e.g. the Pagoda of Fogong Temple in China, Horyu-ji Temple in Japan, Greensted Church in England, and Urnes Stavkirke Stave Church in Norway) or non-degradable carbon when wood is buried in engineered landfills [16–18]. Simultaneously to the HWP carbon being emitted to the atmosphere, well-managed forests regenerate and remove carbon from the atmosphere. Depending on the service lives of wood products and the forest regeneration rate, these events may result in net carbon removals from or additions to the atmosphere. If the input to the HWP carbon pools is larger than the carbon emitted back to the atmosphere, the HWP carbon pools will grow. The longer wood products can delay the release of carbon while forests remove more carbon from the atmosphere, the greater the chance of attaining a net removal from the atmosphere. Theoretically, these HWP carbon pools may eventually reach saturation, at which point the input to the pools will equal the output to the atmosphere. However, it is uncertain when the carbon pools will saturate because the future global demand for wood products is unknown, and some pools, such as landfills can contain carbon that does not decompose [19]. A decrease in the input to HWP carbon pools or a shift towards more short-lived uses may result in the pools saturating more quickly, while an increase in the input or more long-lived uses will delay saturation.

Harvesting and replanting are ways to maintain strong carbon sinks in the forest. The rates of forest growth are strongly age-dependent and, depending on the forest type and other factors, younger trees remove carbon at higher rates than older trees [7, 20]. Several authors have suggested that the best practice from a carbon perspective is to manage the forest sustainably, while continuously supplying society with harvested woody biomass [7, 21–23]. Others have shown that the productions of bioenergy, pulp and paper, and other short-lived products can increase atmospheric carbon for decades [24–28]. Although many factors affect the gains and losses of carbon from forests and harvested wood product carbon pools, sustainable forest management in combination with the use of long-lived wood products can contribute to net removals from the atmosphere. However, careful quantification of the carbon dynamics in ecosystems and in HWP pools should be conducted and compared to a business-as-usual baseline scenario to determine whether a particular forest sector strategy is effective at mitigating climate change [29].

This study is part of the Forest Carbon Management (FCM) Project funded by the Pacific Institute of Climate Solutions (PICS). The FCM project has a broader objective and examines forest sector mitigation options and interactions with climate change [30]. This paper specifically addresses harvested wood biomass utilization and export strategies in BC. More specifically, it focuses on the production, trade and usage decisions along the post-harvest wood products supply chain. The chain begins when harvested biomass is removed from the forest. The ecosystem carbon dynamics, including intensified harvest, conservation, forest regrowth and decay of logging residues left on site are addressed in other analyses (e.g. [30–32]) but are outside the scope of this study.

As a consequence of reduction in growing stocks from the impacts of the Mountain Pine Beetle and large-scale forest fires in recent years, BC is anticipating reduced roundwood harvest for the next few decades [3, 33]. In contrast, population and wood demand are projected to grow for the same period for both BC and its major trading partners [34–37, 49]. On the other hand, ambitious climate targets have incentivized emission reductions in many other sectors such as the aviation and long-range transportation sectors, which are considering the increased use of renewable forest biomass in their greenhouse gas (GHG) emission reduction strategies [38–40]. As a result, forest biomass may become a limited resource that will need to be utilized as efficiently as possible to maximize its contribution to climate change mitigation. Moreover, as BC is considering the development of a bioeconomy focusing on wood first and HWPs value-adding, choices related to the allocation of harvested wood to long-lived products (e.g. in the building sector) or to bioenergy (e.g. biofuels in the transportation sector) can also affect the GHG balance in the forest sector and in other sectors inside and outside of BC.

BC is a net exporter of wood products with 90% of the forest products sold to international markets^2^ in 2018 [11, 41, 42]. Under the current internationally agreed “production approach” reporting rule, Canada is responsible for reporting emissions arising from biomass that was harvested in Canada, regardless of where in the world the emissions occur [43, 82]. Carbon emissions from BC-originated HWPs are strongly affected by their uses in export markets and therefore potential climate benefits vary significantly among different trade and utilization strategies. This paper analyzes the GHG emission consequences of inward- vs outward-focused scenarios and alternative utilization strategies for BC’s HWPs.

## Mitigation scenarios used in this study

The scenarios considered in this paper were designed from a carbon and GHG perspective. For some of the scenarios, the economic or social constraints were intentionally pushed to extreme conditions to provide an indication of a potential range of outcomes (i.e. upper and lower bounds). These theoretical extreme conditions were then adjusted to provide some more realistic scenarios that delivered more meaningful outcomes to the policy community.

The nine scenarios used in this study are summarized in Table 1. The model, parameters and other assumptions are described in “Methods” section.

**Table 1:** Mitigation scenarios used in this study

Mitigation scenarios	Abbreviation
1. Baseline scenario	BASE
2. Theoretical extreme scenarios	
2.1 All harvested biomass manufactured to construction material	ALL_CONS
2.2 All harvested biomass used as feedstock for renewable fuel	ALL_FUEL
3. Inward-focused scenarios	
3.1 Domestic demand increase driven by population	IN_POP
3.2 Increase domestic market share of timber construction	IN_CONS
3.3 Prioritize domestic wood-derived transportation biofuel	IN_FUEL
4. Outward-focused scenarios	
4.1 Prioritize export to US and other markets for construction	OU_CONS
4.2 Prioritize export to China	OU_CN
4.3 Prioritize wood pellets export to EU and Japan for energy	OU_PLTS

### Baseline scenario (BASE)

The BASE scenario provides a business-as-usual baseline. It assumed that the domestic consumption versus trade partitions, and production efficiencies remained constant between 2016 and 2050 [11, 44, 45].

An example of domestic consumption versus trade partitions follows. According to the most recent data, 9% of BC’s total sawnwood production was consumed domestically and 91% was exported [45]. In the BASE scenario, these partition values (i.e. 9% vs. 91%) were assumed to be constant from 2016 to 2050.

As an example of the production efficiencies, average BC forest products manufacturing data indicated that 47.6% of the saw log volume under bark was converted into sawnwood, 38.1% to wood chips and 14.3% to sawdust [11]. In the BASE scenario, these yield partitions (i.e. 47.6%, 38.1% and 14.3%) were assumed to be constant from 2016 to 2050.

All the carbon allocations were estimated using 2016’s production and trade data, assumed to be constant between 2016 and 2050, and configured in the same fashion in the model as the above examples.

### Theoretical extreme scenarios

Theoretical extreme scenarios were designed to demonstrate the theoretical upper- and lower-bounds of the HWPs carbon storage magnitude and the required market demand size. Comparing the results of more realistic scenarios to the outcome of these theoretical boundary scenarios quantifies the degree of achievement and may identify additional opportunities to move closer to the theoretical potentials.

#### All harvested biomass manufactured to construction materials (ALL_CONS)

Wood in constructions generally has longer service lives compared to the other applications.^3^ This scenario assumed that all harvested biomass was manufactured into structural or non-structural products for construction applications. Specifically, sawlogs were sent to sawmills^4^ and plywood mills. Pulp logs were sent to oriented strand board (OSB) mills. Offsite milling residues were sent to medium density fiberboard (MDF) mills and particleboard mills. Aside from the biomass used to produce energy during the manufacturing processes, no harvested biomass was supplied to pulp mills or bioenergy producers.

Sawnwood, plywood and OSB were assumed to be used for structural applications including repair and remodeling, and MDF and particleboard for non-structural components such as cabinets, counters, doors and mouldings.

The ALL_CONS scenario sought to quantify the maximum carbon storage in theory for BC’s wood-based bioeconomy, without considering market, production capacity, financial or technology constraints, and ignoring any leakage. The results can indicate the required market demand in theory for timber construction.

#### All forest biomass as feedstock for renewable fuels (ALL_FUEL)

This scenario assumed that all harvested biomass was used as a feedstock for renewable gasoline, diesel and jet fuel. For simplicity, this study did not assume a technological barrier for these “drop-in” biofuels, the scenario could be applied for any 35-year period from once the technology has become available. As carbon in wood fuels is conventionally treated as instantaneously oxidized, this scenario was equivalent to emitting all the harvested carbon to the atmosphere as CO_2_ in the year of harvest. The mitigation benefit therefore arose only from the substitution effect. This paper examines the biogenic carbon storage and emissions in HWPs and the substitution benefits are out of the scope of this study but will be addressed in future work.

The goal of the ALL_FUEL scenario was to demonstrate the maximum theoretical carbon emissions from HWPs as well as determining whether the projected harvest would be sufficient to fulfill the energy demand of BC’s transportation sector.

### Inward-focused scenarios

BC has long been a major net exporter of wood products and consequently the role of BC’s HWPs in emission reductions is expected to be strongly affected by foreign policies and behaviors. The inward-focused scenarios were designed to address the potential significance of the domestic market from a carbon perspective. They are also examples of BC’s increasing effort to export technology, management and innovation, rather than just forest products [46–48]. These scenarios prioritized domestic consumption over international trade. Overall HWP exports decreased but the proportional allocation to different jurisdictions remained as in the BASE scenario.

#### Domestic demand increase driven by population (IN_POP)

Canada’s population is projected to increase over the coming decades. In general, HWP demand correlates with population size [35, 36, 49]. This scenario assumed that the demand for domestic HWPs increased in proportion to population growth [34]. The IN_POP scenario was the basis for all inward- and outward-focused scenarios, meaning that the increased domestic demand for various HWPs due to population growth had to be satisfied first, unless indicated otherwise.

#### Increase domestic market share of timber construction (IN_CONS)

Housing completions, in general, follow the trend of household formation [35]. This scenario therefore assumed that the domestic demand of residential dwellings increased in proportion to household growth. Non-residential buildings also usually increase as communities grow larger. Consequently, the domestic demand of the non-residential buildings was assumed to also increase proportionately with household growth.

In Canada, roughly 30% of the residential dwellings and 10% of the non-residential buildings are built with wood, although these shares are larger in BC at approximately 50% of residential and 25% for non-residential [50–52]. For comparison, wood accounts for 97% of all framing material and 64% of floor construction in the US residential dwellings [53]. Given the similarities between the US and Canadian market and construction culture, the domestic timber construction market has some significant potential that has not been realized. In addition, the Wood First program, the international promotion of mass timber tall wood buildings, and changes in the building codes should also strengthen future market growth of timber constructions [54–56]. The IN_CONS scenario assumed that the domestic timber construction market share doubled between 2017 and 2050.

#### Prioritize domestic wood-derived transportation biofuel (IN_FUEL)

The transportation sector contributed 40% of BC’s emissions and 24% of Canada’s emissions [57, 58]. There have been substantial investigations into wood-derived drop-in biofuels in BC to reduce transportation emissions [38–40, 59]. While sawlogs are normally considered too valuable to be used directly as a bioenergy feedstock, milling residues and pulp logs are suitable [60]. Bark and 6–10% of the milling residues are already burnt onsite for energy [11].

The IN_FUEL scenario assumed that all pulp logs and offsite milling residues were used as feedstocks for renewable gasoline, diesel and jet fuel, while sawnwood and plywood production, as well as their market partitions, remained the same as the IN_POP scenario.

### Outward-focused scenarios

Under the circumstances of a projected declining harvest and a growing population [33, 34], the outward-focused scenarios assumed that BC’s forest sector fulfilled the domestic demand in the IN_POP scenario first and then allocated the remaining products to foreign regions. Three allocation options were explored.

#### Prioritize export to US and other markets for construction products (OU_CONS)

The United States imports 60% of BC’s solid and composite wood products, and is by far BC’s largest importer [41, 45]. China imports 20% of BC’s solid and composite wood products and 50% of BC’s pulp and paper products, and is BC’s second largest importer [45, 61]. The EU imports over 80% of BC’s wood pellets and smaller proportions of other products [45]. The US uses a large proportion of wood as construction material and the timber construction market share is one of the highest in the world [53, 62]. China, on the other hand, mostly uses wood for short-lived applications such as concrete casing and packaging [63]. The EU mostly combusts BC’s pellets for heat and electricity [60].

Despite the diverse utilization practices, these markets all have a size that can potentially consume a great amount of BC wood as construction materials. The US has a construction market that is 7 times larger than Canada’s [64]. China has one that is over 30 times larger [65], whereas the EU has one that is about 8 times larger [66, 67]. Studies have indicated that replacing concrete and steel with engineered wood products in constructions can reduce emissions [68–70].

The OU_CONS scenario assumed that the domestic demand in the IN_POP scenario was fulfilled first, then the biomass remaining was manufactured into construction purpose products, exported and used as intended. Refer to the section of the ALL_CONS scenario for a description of construction purpose products.

#### Prioritize export to China (OU_CN)

China replaced Japan and became the second largest importer of BC wood products after the Global Financial Crisis in 2008 [45, 61, 71]. The principal products imported by China are raw logs, sawnwood and chemical pulp. The end uses of BC’s wood in China are mostly in the shorter-lived categories such as paper, concrete casing, shipping and packaging [63].

The OU_CN scenario assumed that the domestic demand in the IN_POP scenario was fulfilled first, then the biomass remaining was manufactured into products in proportion to the business-as-usual end-use pattern in China, exported to China and used as intended.

#### Prioritize wood pellets export to EU and Japan for energy (OU_PLTS)

The European Union has set targets to increase the share of renewables in energy consumption to 20% in 2020 and 27% by 2030 [72]. It has also been estimated that in order to achieve a low-carbon economy by 2050, the EU countries would need to triple their current level of bioenergy consumption and the feedstock would largely come from the agricultural and forestry sectors [73]. One of the options being investigated was to import wood pellets primarily from Canada and the US [60, 74].

The European Union has already been the largest consumer of BC wood pellets with an import share of over 80% [45]. Their pellet demand will continue to rise if they decide to rely heavily on wood-based bioenergy.

Another major wood pellets importer is Japan. Its import has increased ten-fold between 2012 and 2018 [75]. A long-term contract was signed between BC and Japan in 2019 to export 100,000 tonnes of wood pellets annually [76].

The OU_PLTS scenario assumed that the domestic demand in the IN_POP scenario was fulfilled first, then the biomass remaining, including sawlogs, was manufactured into wood pellets and exported to EU countries and Japan.

## Results

This section describes the HWPs biogenic carbon emission and storage implications of the nine scenarios outlined above using “Methods” that are further explained after the “Conclusions”.

In all scenarios, BC’s roundwood harvest gradually declined from 60 MtCO_2_e yr^−1^ in 2016 to 51 MtCO_2_e yr^−1^ in 2050, with a cumulative transfer of carbon from forests to bioeconomy of 1900 MtCO_2_e.

This study did not explore alternative uses of bark. Bark was allocated to hog fuel in the model. Emissions from bark combustion of 245 MtCO_2_e between 2016 and 2050 (on average 7.0 MtCO_2_e yr^−1^) were the same for all 9 scenarios. Bark emissions were not included in the following figures to avoid dilution of the impact of different trade and consumption strategies and to improve readability. Under bark volume data were used in accordance with FAOSTAT and BC mill surveys [11, 77].

Inherited emissions, which are carbon losses from HWPs manufactured from roundwood harvested prior to 2016, were also not reported below because this study examined future mitigation scenarios and the inherited emissions were the same for all scenarios.

### Baseline scenarios (BASE)

**Fig. 1:**
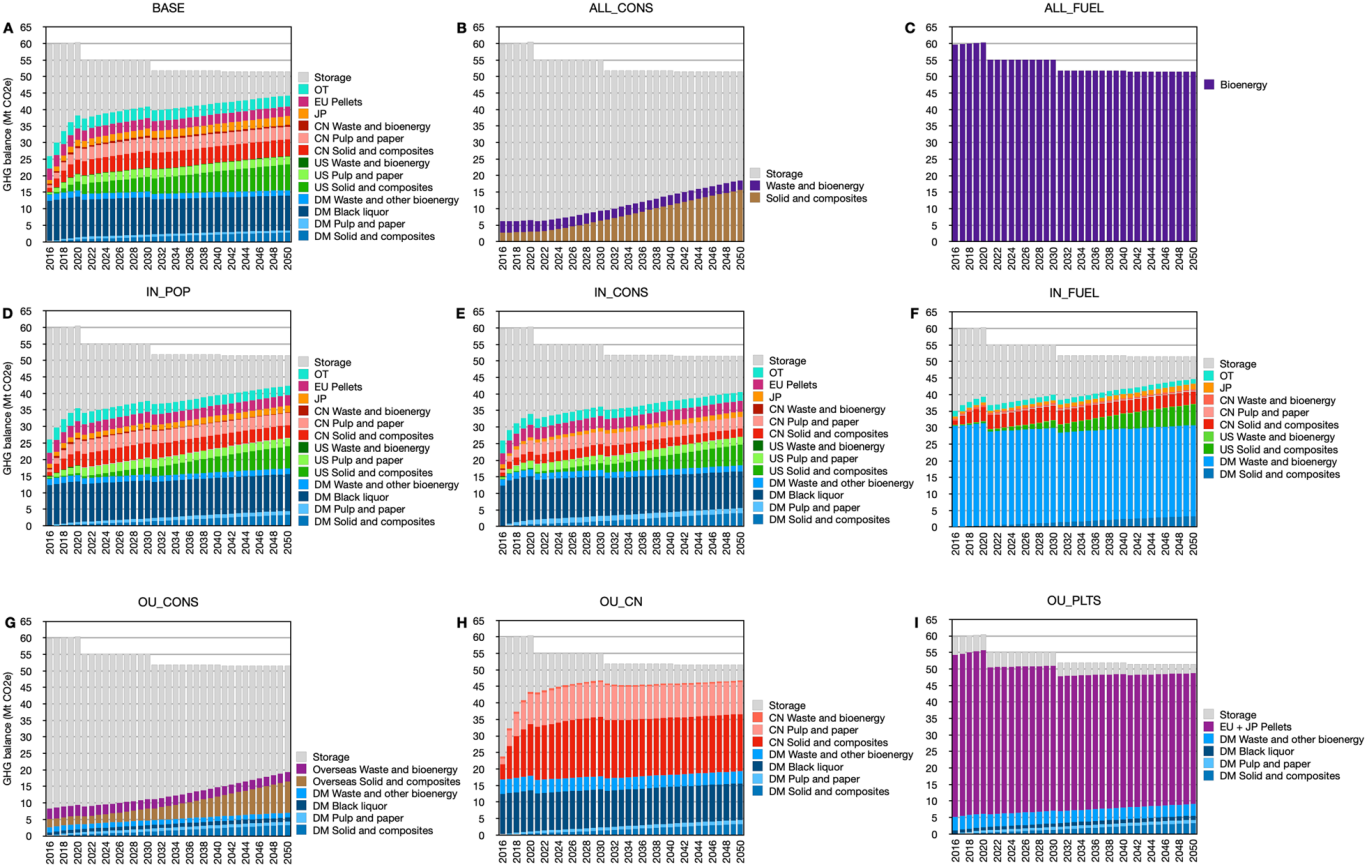
Annual GHG balance of HWPs originated from BC between 2016 and 2050 in the **a** BASE, **b** ALL_CONS, **c** ALL_FUEL, **d** IN_POP, **e** IN_CONS, **f** IN_FUEL, **g** OU_CONS, **h** OU_CN and **i** OU_PLTS scenarios. Emissions from bark, 245 MtCO_2_e between 2016 and 2050 or on average 7 MtCO_2_e yr^−1^, are not included. DM: domestic; US: United States; CN: China; JP: Japan; EU: European Union; OT: other jurisdictions; Storage: the amount added to the net carbon storage (i.e. annual harvests minus annual emissions from products harvested since 2016). All color bars except grey correspond to biogenic emissions from HWPs

In the BASE scenario, 74% of the carbon in HWPs produced after 2016 (roughly 1400 MtCO_2_e or on average 40 MtCO_2_e yr^−1^) would be emitted to the atmosphere over the period of 2016–2050, with the remainder 26% (approximately 490 MtCO_2_e) staying in the HWPs (Fig. 1A). The annual emissions of carbon from wood harvested since 2016 increased over time from 26 MtCO_2_e yr^−1^ in 2016 to 44 MtCO_2_e yr^−1^ in 2050. The rate of carbon accumulation in the pool slowly decreased over the study period from 33 MtCO_2_e yr^−1^ in 2016 to 7.0 MtCO_2_e yr^−1^ in 2050. The initial increase of emissions from 2016 to 2020 was mainly due to emissions within China and the US, and reflects that inherited emissions from pre-2016 harvest are not included in this study. The Chinese emissions largely arose because the weighted average service life of wood in China was about 6 years. The amount emitted in the US during this period mainly came from wood used in industrial production such as pallets, concrete casings, and paper products. These applications had short service lives. China imported less than half of the roundwood equivalents than the US, but the emissions from wood harvested since 2016 were roughly the same as those in the US because of China’s short-lived wood utilization practices. Annual emissions increased steadily and slowly from solid wood and wood composites within the US and Canada. Geographically, the largest emissions occurred within Canada, with 67–85% of the domestic emissions originating from burning black liquor in the kraft pulp recovery boiler annually between 2016 and 2050.

### All forest biomass manufactured to construction materials (ALL_CONS)

If all of BC’s harvested biomass were used for construction, there would be a substantial delay in carbon release (Fig. 1B). In this scenario, the annual carbon storage ranged from 53 MtCO_2_e yr^−1^ in 2016 to 33 MtCO_2_e yr^−1^ in 2050. The carbon stored in these structures would slowly release back to the atmosphere. In 2050, 79% (1500 MtCO_2_e) of the carbon harvested since 2016 was still remaining in products. Annual emissions ranged from 6.1 MtCO_2_e yr^−1^ in 2021 to 19 MtCO_2_e yr^−1^ in 2050. There were some immediate carbon releases of approximately 3.1 MtCO_2_e yr^−1^ due to unrecovered losses and residue combustion for processing energy.

### All forest biomass as feedstock for renewable fuels (ALL_FUEL)

In this scenario, all harvested carbon was released back to the atmosphere in the year of harvest because it was used as feedstock for renewable gasoline, diesel and jet fuel (Fig. 1C). The quantity emitted equaled the harvest amount which averaged 54 MtCO_2_e yr^−1^ for the period 2016–2050. There was no carbon storage.

### Domestic demand increase driven by population (IN_POP)

The IN_POP scenario followed a similar HWPs emission trajectory to the BASE scenario (Fig. 1D). The annual emissions ranged from 26 MtCO_2_e yr^−1^ in 2016 to 42 MtCO_2_e yr^−1^ in 2050, slightly less than the BASE scenarios. The largest domestic emission source remained black liquor combustion. Saw log exports reduced from 10 to 2% and all the pulp logs were consumed domestically. The largest importer of BC’s raw logs continued to be China and the HWPs service lives in Canada were longer than in China, which consequently reduced the emissions. A small proportion of OSB and graphics paper was shifted to be consumed domestically, but that had only minor effects on emission reductions.

### Increase domestic market share of timber construction (IN_CONS)

In this scenario, raw log exports in the BASE scenario were shifted to be processed for the domestic building sector. Some exported sawnwood, plywood, OSB and MDF were also shifted to domestic construction. The general emissions trajectory of this scenario is similar to the BASE and IN_POP scenarios. The annual emissions ranged from 26 MtCO_2_e yr^−1^ in 2016 to 41 MtCO_2_e yr^−1^ in 2050 (Fig. 1E). The emissions from the domestic regions were slightly higher than the BASE and IN_POP scenarios mainly due to additional renovation and demolition. However, the utilization and trade change reduced emissions from the foreign regions, especially in China, resulting cumulatively in a 10% net total reduction compared to the BASE scenario.

### Prioritize domestic wood-derived transportation biofuel (IN_FUEL)

In this scenario, BC produced transportation biofuels using pulp logs and milling residues while keeping the sawlog utilization the same as the IN_POP scenario. The annual emissions ranged from 35 MtCO_2_e yr^−1^ in 2016 to 44 MtCO_2_e yr^−1^ in 2050 (Fig. 1F). A proportion of the carbon previously emitted from short-lived end uses in the export markets was now emitted from the biofuels consumed in the domestic market.

Although the domestic emissions increased, the produced biofuels were expected to displace conventional transportation fuels and achieve emission reductions. The climate benefits of drop-in biofuels have been studied by several life cycle assessments (LCA) (e.g. [78–81]). A quantification of the substitution benefit under the IN_FUEL scenario will be addressed in the future.

### Prioritizing exports to US and other markets for construction (OU_CONS)

In this scenario, exported biomass was manufactured into and used as construction materials. The annual emissions ranged from 8 MtCO_2_e yr^−1^ in 2016 to 19 MtCO_2_e yr^−1^ in 2050 (Fig. 1G). By 2050, 74% (1400 MtCO_2_e) of the carbon harvested since 2016 had been added to the HWP pool under this scenario.

### Prioritizing exports to China (OU_CN)

Wood in China is primarily used for short-lived purposes [63]. In this scenario, the annual emissions increased, compared to the BASE scenario, and ranged from 24 MtCO_2_e yr^−1^ to 47 MtCO_2_e yr^−1^ (Fig. 1H). However, the single year emissions in 2016 were lower than those in the BASE scenario because, in the OU_CN scenario, BC did not produce wood pellets for export to the EU. Instead, it produced more solid and composite wood products for short-lived applications and kraft pulp for the Chinese market. The half-lives of these products in China were approximately 2 to 3 years [63, 82]. Therefore the emissions started to surpass the BASE scenario in 2017. Manufacturing more kraft pulp for China also resulted in increased emissions from black liquor within BC.

### Prioritizing exports to EU and Japan for energy (OU_PLTS)

The EU could potentially consume up to approximately 26 Mt of wood pellets from Canada [60]. Japan was projected to consume 6.8 Mt of wood pellets by 2025 [75, 83]. In this scenario, BC produced about 21 Mt of wood pellets. The annual emissions decreased from 54 MtCO_2_e yr^−1^ in 2016 to 49 MtCO_2_e yr^−1^ in 2050 due to the declining harvest (Fig. 1I). Carbon storage only occurred in the domestic regions. There was no foreign carbon storage benefit because wood pellets were considered to be emission in the year of harvest. The associated emissions are reported in BC’s GHG inventory, while the avoided fossil fuel emission reductions are reported by the countries that burn the pellets [29].

### Comparisons

**Fig. 2:**
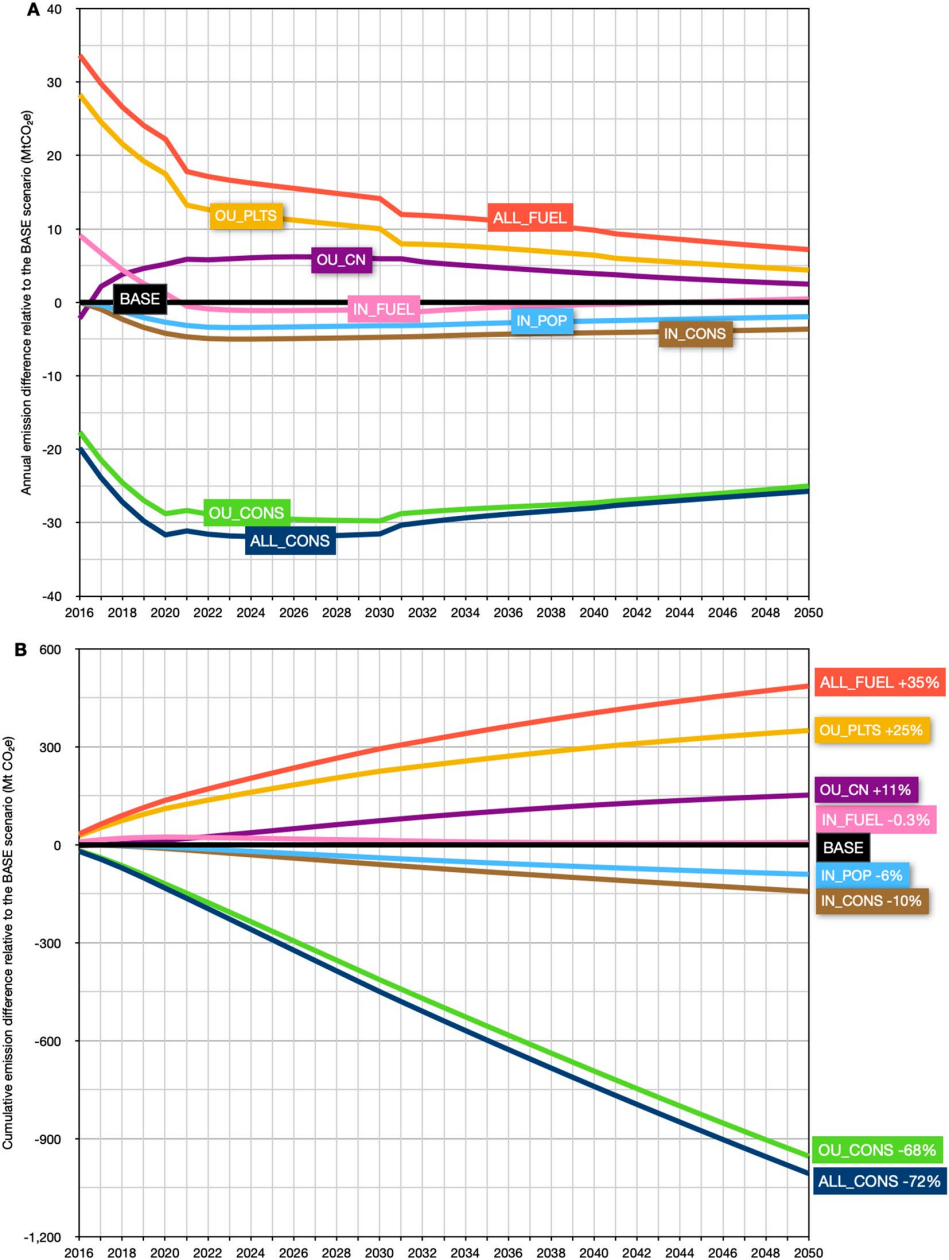
**a** Annual and **b** cumulative emission differences of all mitigation scenarios relative to the BASE scenario. The black line at y = 0 indicates the BASE scenario. All scenario names are defined in Table 1

Figure 2A summarizes the annual emission differences of all mitigation scenarios relative to the BASE scenario and Fig. 2B shows the cumulative differences. Scenarios that prioritized short-lived HWP utilizations emitted more than the BASE scenario. Scenarios that promoted HWPs as long-lived construction materials achieved greater mitigation benefits. The inward-focused scenarios emitted less than the BASE scenario. The gaps of annual emissions among all scenarios were narrowing over time as carbon was slowly emitted back to the atmosphere. This was also observed in Fig. 2B as the slopes of the curves were declining over time, however the magnitude of cumulative differences were substantial.

**Fig. 3:**
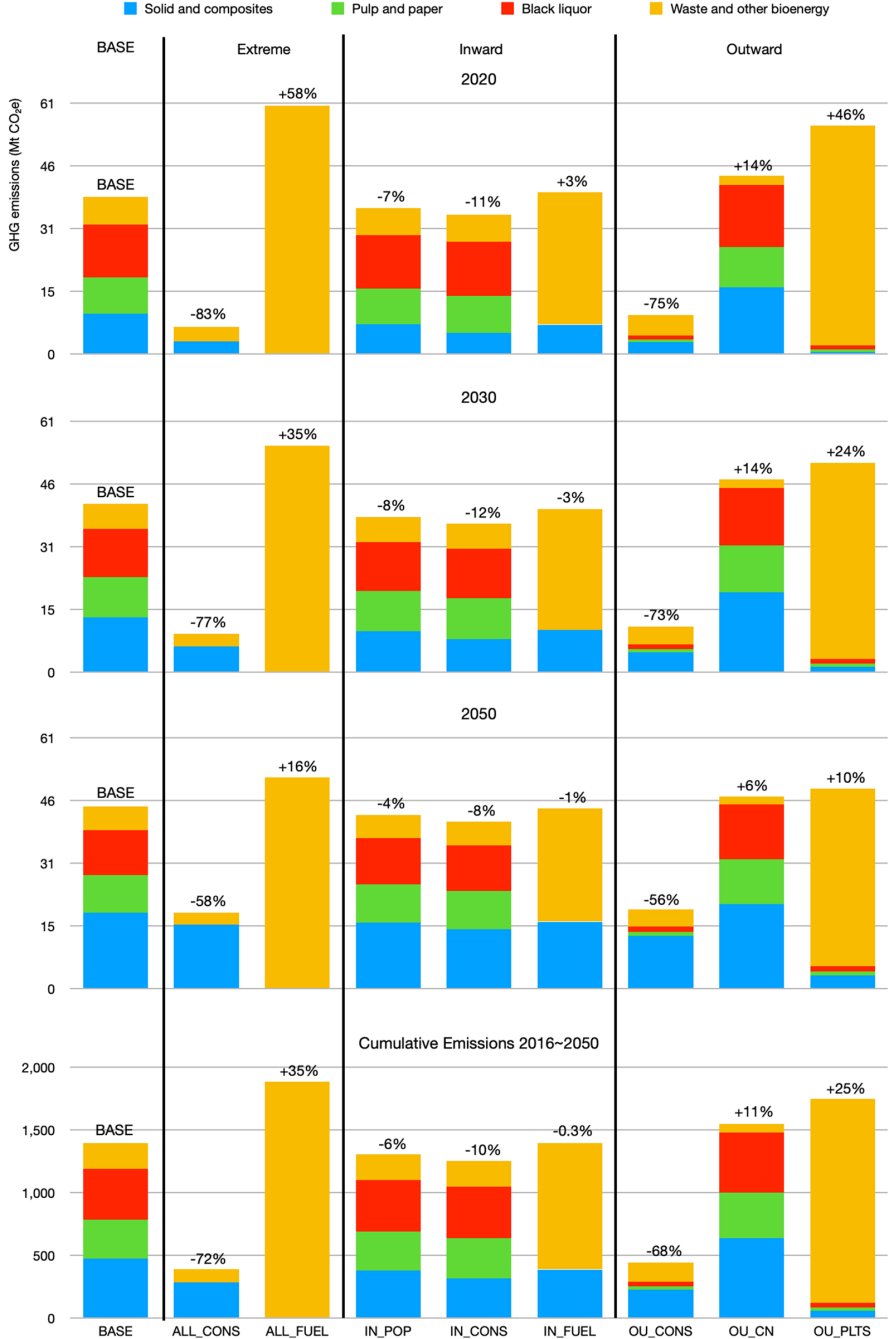
A comparison of the emission consequences at year 2020, 2030, 2050 and cumulative emissions from 2016 to 2050. Emissions from bark, 245 MtCO_2_e between 2016 and 2050 or on average 7 MtCO_2_e yr^−1^, are not included. The ± percentage numbers on top of the bars are emissions changes compared to the BASE scenario

The detailed emission consequences of each scenario can be more easily compared by evaluating time points at year 2020, 2030, 2050 and by aggregating the emissions from 2016 to 2050 (Fig. 3).

The ALL_CONS scenario achieved the lowest emissions among all scenarios studied. This scenario generated the lowest emission possible in theory from BC’s HWPs, because all the harvested forest biomass was allocated to wood products processing facilities to produce structural and non-structural products for construction. Therefore, except for onsite energy use of wood chips, sawdust and shavings, most carbon was stored in construction. Cumulatively, this scenario emitted 391 MtCO_2_e (or on average 11 MtCO_2_e yr^−1^) which was 72% less than the 1,400 MtCO_2_e (or on average 40 MtCO_2_e yr^1^) emitted by the BASE scenario (Fig. 3 cumulative). The emissions of the ALL_CONS scenario gradually increased over time from 6.5 MtCO_2_e yr^−1^ in 2020 to 18.6 MtCO_2_e yr^−1^ in 2050, as the carbon stored in timber construction was slowly emitted to the atmosphere due to renovation and demolition (blue bars in Fig. 3 2020, 2030 and 2050). Although the emissions almost tripled between 2020 and 2050, they were still much lower than the average 40 MtCO_2_e yr^−1^ emissions in the BASE scenario.

Achieving this lowest emissions scenario would require the use of BC wood to construct annually 10,000 Brock Commons Tallwood Houses,^5^ equivalent to a total floor area of 154 million m^2^ yr^−1^. However, at the time of this study, BC wood was used to construct only 30 million m^2^ yr^−1^: 3 million m^2^ yr^−1^ domestically and 27 million m^2^ yr^−1^ abroad [50–53, 86, 87]. For comparative purposes, in 2016, China had annual residential housing starts of 2 billion m^2^ yr^−1^ [65]; US had 177 million m^2^ yr^−1^ [88]; Canada had 32 million m^2^ yr^−1^ [67]; BC had 8 million m^2^ yr^−1^ [86, 87]; the UK had 3 million m^2^ yr^−1^ [89]. The wood frame market shares of single-family houses and low- to mid-rise buildings in the US and Canada are already relatively high [50–53], but further potential exists for high-rise residential buildings, non-residential buildings and public infrastructure. The timber construction market share in China is low but the authorities have shown interest in exploring options to increase this value [49]. It is noteworthy that even a small market penetration in China, such as 10%, would mean an achievement of the demand required in the ALL_CONS scenario.

While the ALL_CONS scenario generated the lowest possible emissions, the ALL_FUEL scenario resulted in the largest possible emissions from BC’s HWPs. These two scenarios provided the theoretical upper and lower emission bounds. In the ALL_FUEL scenario, the emissions gradually declined over time from 60 MtCO_2_e yr^−1^ in 2020 to 51 MtCO_2_e yr^−1^ in 2050 in accordance with the harvest projections. Cumulatively, this scenario emitted 35% more than the BASE scenario.

The ALL_FUEL scenario also helps determine whether there is enough forest biomass to satisfy BC’s energy demand in the transportation sector. The CleanBC plan sets a biofuel production target of 650 million liters by 2030 [90]. Our calculation indicates that there is ample forest biomass in BC to achieve this target (Additional file 3 Renewable transportation fuel calculation). However, this biofuel target is only 8% of the fuel demand of BC’s transportation sector. In order to satisfy the fuel demand of the province’s entire transportation sector, BC would need to consume all of its harvested biomass and even then fuel production would fall about 1% short. For further context, BC’s demand is only 13% of Canada’s transportation fuel requirements.^6^ Using all harvested biomass for fuels is not climate effective, it would not be economically viable, and it would likely not be socially acceptable.

The IN_POP scenario achieved a cumulative 6% decrease in emissions compared to the BASE scenario. With the exception of US (34 years), the weighted average service lives of HWPs in Canada (32 years) are generally longer than those of China (6 years), Japan (26 years) and other importers, which led to the emission reductions observed. The emission reductions were higher in 2020 at 7% but declined to 4% in 2050. This was because renovations usually occur at around 30 years of service life. Therefore, slightly more carbon from solid and composite wood products was released after 2045 than in the BASE scenario (Fig. 1A, D). The increased domestic consumption of solid and composite wood products also increased the amount of milling by-products and residues, which led to slightly increased downstream emissions from waste, bioenergy and pulp and paper.

The IN_CONS scenario achieved the lowest emissions among the three inward-focused scenarios. Cumulatively, this scenario achieved a 10% emission reduction compared to the BASE scenario. The emission reduction was higher in 2020 at 11% and declined to 8% in 2050. This trajectory is the same as the IN_POP scenario because HWPs for renovation purposes were approaching their end-of-life around 2045.

Unexpectedly, the IN_FUEL scenario did not increase the HWP emissions. Compared to the BASE scenario, the emissions were only higher for the period before 2020 and became lower thereafter (Fig. 1F). More specifically, there was a 3% emission increase in 2020 and a 1% decrease in 2050 (Fig. 3). The cumulative emissions were actually 0.3% less than the BASE scenario. This was because of the configuration of the scenarios. In the IN_FUEL scenario, sawlogs were primarily used to produce sawnwood and plywood for both domestic and international markets, similar to the IN_POP scenario. Pulp logs, milling by-products and residues were allocated for renewable fuel production. Increased domestic demand driven by population growth shifted some sawnwood and plywood from foreign markets to be consumed domestically, similar to the IN_POP scenario. The emission reduction caused by this shift slightly outweighed the increased emissions associated with biofuel combustion.

The biofuel produced in the IN_FUEL scenario can cover 50% of BC’s transportation demand or 7% of Canada’s. Since sawlogs are not normally used as a feedstock for biofuels, this is the largest output that is theoretically feasible for BC without harvesting additional feedstocks from non-merchantable biomass, for example using biomass currently burned as post-harvest residues [30].

The OU_CONS scenario resulted in a cumulative emission of 444 MtCO_2_e, which was a reduction of 68% compared to the BASE scenario. The emission reduction is higher in 2020 at 75% and declined to 56% in 2050 due to renovation. This scenario required trading partners to build the equivalent of 9,000 Brock Commons Tallwood Houses per year using BC wood. That floor area is approximately 75% of the US housing starts or 8% of the Chinese housing starts [64, 65].

The markets for short-lived applications in China and wood pellets in the EU and Japan are large but generated 11% and 25% more emissions, respectively, compared to the BASE scenario. Notably, emissions were higher in these scenarios than the IN_FUEL scenario, because the IN_FUEL scenario assumed that sawnwood and plywood were predominantly exported to the US for relatively longer-lived uses.

## Discussion

The results have shown that the utilization and trade of harvested forest biomass can have a significant influence on BC’s future biogenic emissions. More specifically, scenarios that extended the service lives of HWPs (i.e. ALL_CONS, IN_POP, IN_CONS and OU_CONS) resulted in lower emissions than those that focused on short term uses (i.e. ALL_FUEL, IN_FUEL, OU_CN and OU_PLTS). The largest cumulative emission difference of 1,500 MtCO_2_e occurred when the two theoretical boundary scenarios, ALL_CONS and ALL_FUEL, were compared (Fig. 3). However, these extreme scenarios are not an indication of the future emissions. The more practical scenarios using population and market capacity bases produced a lower cumulative emission difference of 493 MtCO_2_e between the IN_CONS and OU_PLTS scenarios. For comparison, BC’s emissions from HWPs not accounting for the compensating sinks in forests and from all sectors except forestry are about 40 MtCO_2_e yr^−1^ and 60 MtCO_2_e yr^−1^, respectively [57]. The IN_CONS scenario resulted in 36 MtCO_2_e yr^−1^ emissions from HWPs in 2030 which is approximately 3 MtCO_2_e yr^−1^ lower than the 2007 level [57]. This more practical scenario that promoted wood as a low carbon building material compared well with the emission reductions target in the CleanBC plan [90] which specified a 2 MtCO_2_e yr^−1^ emission reduction by 2030 in the building sector. These reductions are achieved only through increased carbon retention in wood products in use, while additional emission reductions through the substitution of emissions-intensive building materials will occur but were not included here. Therefore, although the long-term strategy that focused on wood construction within Canada resulted in a smaller mitigation benefit than the OU_CONS scenarios, it can still contribute meaningfully to BC’s emission reduction goals, and can be achieved without behavior changes in export markets.

Moreover, the construction-focused scenarios may create an additional climate benefit. This paper treated landfilled woody biomass as instantaneous oxidation (see “Methods” section), which is consistent with UNFCCC adopted decisions for the Kyoto Protocol [43] and Canada’s National GHG Inventory Report [93]. However, in reality, some of the carbon in post-consumer HWPs, especially retired building materials, may be stored in landfills for an extended period of time. Countries such as the US and Australia have included the landfill dynamics in their inventory reporting [94, 95]. These reporting improvements may reduce eventual emissions from timber constructions and may increase the benefits of the construction-focused scenarios. However, this conclusion depends heavily on the management and use of methane emissions from landfills. Technological innovation in the wood products supply chain can also provide additional climate benefits, but this topic was beyond the scope of this study (Table 2).

**Table 2:** A summary of the scope

	Included	Not included
Alternative forest management and wood harvest strategies		•
Alternative HWP utilization and trade strategies	•	
Jurisdiction-specific wood allocations	•	
Future technological innovation		•
Fossil fuel emissions associated with upstream, manufacture and transportation		•
Biogenic emissions from HWP end uses	•	
Biogenic carbon retention in HWP end uses	•	
Substitution effects of HWP end uses		•
Biogenic carbon dynamics of HWP in landfills		•

The domestic market only consumes about 15% of BC’s harvests [11, 44, 45] and the inward-focused strategies demonstrated mitigation potentials that were limited by the size of the domestic market. In general, the inward scenarios varied only by a ± 5% difference. The foreign markets for HWPs are larger, however the emissions varied substantially depending on how and where the exported wood was used (cf. OU_CONS, OU_CN and OU_PLTS in Fig. 3). The mitigation analysis indicated that, from the perspective of GHG emission reduction targets only, BC was better off consuming all harvested biomass domestically for various product demands including transportation biofuels, and only exporting wood for long-lived purposes, rather than short-lived applications. In the past decade, the share of BC’s lumber in the US timber construction market has averaged 17% with the highest being 20% [49]. The US construction market is unlikely able to take all the exported wood outlined in the OU_CONS scenario. Therefore, if BC were to move to this more timber-construction-prioritized bioeconomy, BC would have to rely on enhancing domestic timber construction market share, accessing the US market, and penetrating new international markets. China is the only other market with sufficient scale. The Canada-US Softwood Lumber Dispute and the Sino-Canada trade tensions may be major roadblocks for the realization of this bioeconomy scenario. If there is not sufficient demand in the domestic and foreign construction markets, this study showed that it may be better for BC to pursue wood-derived transportation biofuel investments and substitute fossil fuels in the domestic market than to export to jurisdictions with short-lived wood product utilization practices. This was demonstrated by the IN_FUEL scenario resulting in lower emissions than the BASE, OU_CN and OU_PLTS scenarios (Fig. 3). Although the IN_FUEL scenario was a hypothetical scenario, since OSB, MDF and paper products were not produced, it revealed that exporting wood pellets, raw logs and kraft pulp were not the best utilization of BC’s forest resources from a biogenic emissions perspective.

Another example of how HWPs may influence future emissions was shown by a consideration of the black liquor emissions from kraft pulp manufacturing. Producing kraft pulp from BC’s harvested biomass generated a significant amount of black liquor which emitted on average 11 MtCO_2_e yr^−1^ and cumulatively 402 MtCO_2_e over the study period (Fig. 3: red bars in the BASE scenario). Combusting black liquor reduces kraft pulp mills’ dependency on fossil energy sources and recovers process chemicals. However, the resulting paper product is still short-lived. Prioritizing pulp export will increase BC’s emissions (OU_CN). Pulp mills in BC may claim energy self-sufficiency and emission reduction achievements by using black liquor and wood residues to generate electricity under the assumption of carbon neutrality of bioenergy [96, 97]. However, from an atmospheric carbon perspective wood-based bioenergy is at best “carbon lean” instead of “carbon neutral” [98]. Moreover, BC’s electricity can largely be generated by hydropower which has a lower climate impact. Substituting hydropower with black liquor combustion provides little climate benefit, if any. On the other hand, black liquor can be converted into liquid transportation fuels and displace fossil fuels [99, 100]. Notably, less than 10% of the black liquor emissions arose from pulp and paper produced for domestic consumption. Over 90% of those emissions were associated with the production of pulp and paper for export.^7^ That said, BC’s pulp and paper sector contributes 1.5 billion dollars to the province’s GDP on an annual basis and employs eight thousand people [101–104].

Similarly, if BC were to develop a bioeconomy policy that only would make long-lived construction products, there would be a significant impact on international trade and this bioeconomy could fail economically. A forest industry with a diverse product portfolio provides resilience to the province’s economy and communities. The prosperity of human society relies on wood to provide more than just long-term uses such as housing. Even if these social and economic implications were ignored, there would be substantial emission leakages if forest biomass were limited to long-term applications. If the world demands wood fibre for paper manufacturing, it is going to be manufactured somewhere. Restricting pulp production and exports in BC will not eliminate global black liquor emissions.

Wood product markets are demand-driven and may have conflict with environmental outcomes. Therefore, BC may wish to develop a utilization hierarchy to facilitate the achievement of the optimum emission reduction, while achieving the desired economic outcomes. A utilization hierarchy would define a priority cascade based on the mitigation analysis results and advise the policy community to develop a hierarchical carbon incentive system. Such a system would coexist with other market-drivers. However, in order to develop such a utilization hierarchy, mitigation analyses should go beyond biogenic emissions. Substitution effects of wood-based bioenergy compared to wood as a construction material should also be investigated.

## Limitations and future work

This research compared various harvested wood products mitigation strategies from a carbon perspective with consideration of market size and access. The goal was to be descriptive rather than prescriptive. Although the overall conclusions are believed to be widely applicable, there are some limitations as highlighted in Table 2.

1. Alternative forest management and wood harvest strategies were not the focus of this study. These have been addressed by others [cf. 31,32].
2. Fossil fuel emissions associated with upstream, production and transportation of HWPs are important for comparing different mitigation strategies and estimating the substitution benefits of various HWP end-uses. This paper examines the biogenic carbon storage and emissions in HWPs. Substitution benefits were beyond the scope of this study but will be addressed in future work. The substitution effects of HWP are usually calculated using displacement factors determined from the results of comparative life cycle assessments which factor in fossil emissions. Integrating the fossil emission assessments directly into the model is also possible [105, 106] and would expand the scope and accuracy of the estimated climate impact of the modeled mitigation strategy.
3. The carbon dynamics of HWP in landfills and the resulting emissions of CO_2_ and methane are complex and were beyond the scope of this study. Given its potential for long-term storage, this topic will be a focus of our future work.
4. This study’s scenarios placed relatively more weight on two main end uses, timber construction and wood-derived transportation biofuel as outlined in the CleanBC plan [90]. Other mitigation options of novel technologies such as cellulose nanomaterials, or conventional technologies such as wood pellets replacing coal and natural gas in heat and power production, were not examined. In reality, the future structure of BC’s wood-based bioeconomy will likely be a dynamic mix of conventional and novel technologies.
5. The assessment of the mitigation strategies developed in this research was limited to the magnitudes of emission reduction benefits and market access. Developing viable mitigation solutions will also require socioeconomic analyses.

## Conclusions

British Columbia has substantial forest resources which provide opportunities for GHG mitigation that few other jurisdictions possess. Allocations of wood flows to long-lived or short-lived products or bioenergy had a substantial impact on BC’s future emissions profile. The mitigation analysis revealed that from a choice between inward- and outward-focused bioeconomy and a biogenic carbon emission reduction from harvested wood products perspective, it was better for BC to consume the harvested biomass within Canada and only export those harvested wood products that would be used as long-term construction materials, such as structural lumber, engineered timber products and wood composites for cabinets, walls and countertops. Using this approach, BC could achieve an emission reduction from harvested wood products of 68% compared to the business-as-usual baseline. For this strategy to be successful, BC needs to have access to the US and Chinese construction markets, or the mitigation outcomes will be constrained by the limited size of the domestic market. However, a bioeconomy strategy that relies totally on exports of long-lived timber products will have a significant impact on international trade, and could fail economically as pulp, bioenergy and other short-lived uses of wood have their exports restricted. The more practical scenario that focused on increasing wood construction market shares within Canada had a small but politically and environmentally meaningful contribution to the province’s low carbon building plan with a 3 MtCO_2_e yr^−1^ emission reduction from harvested wood products by 2030 and 4 MtCO_2_e yr^−1^ by 2050. These reductions are achieved only through increase in carbon retention in wood products, while additional emission reductions through the substitution of emissions-intensive building materials are not included here. Because a demand driven bioeconomy can have conflicts with the desired environmental outcomes, BC may wish to adopt a hierarchical climate change mitigation incentive system that can coexist with the other market-drivers to facilitate the achievement of optimal emission reductions. In addition to this study, which conducted mitigation analyses from a biogenic emissions perspective, the development of this system would require a comprehensive understanding of the substitution effects and landfill dynamics of wood constructions and wood-derived biofuels.

## Methods

This section describes the HWP mitigation analysis (MitigAna) model used in this study, as well as the study period, data sources, tools, simulation steps, and the biomass supply assumptions used for various scenarios.

### The MitigAna model

**Fig. 4:**
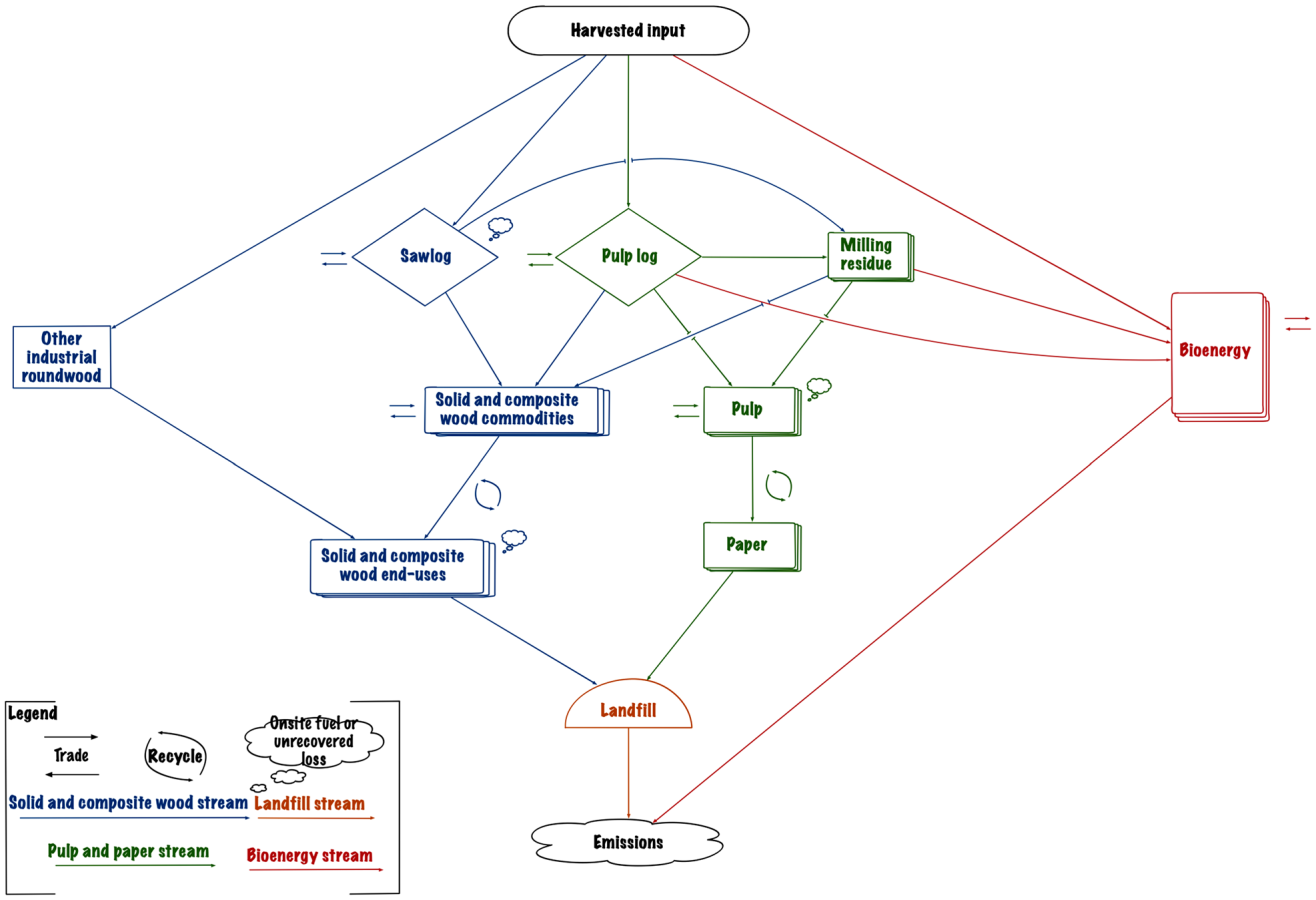
Conceptual harvested wood products carbon flow in the MitigAna model

The MitigAna model is a harvested wood products carbon dynamics model designed to simulate the fate of wood carbon for scenario-based *mitigation analysis*. MitigAna was implemented using the Carbon Budget Modeling Framework for Harvested Wood Products (CBMF-HWP) which is a software system developed by the Canadian Forest Service (Mark Hafer and Michael Magnan, personal communication). During model development, we also made reference to two other models based on CBMF-HWP, the NFCMARS-HWP model [93] and the BC-HWPv1 model [107], and modeling principles in Brunet-Navarro et al. [108]. A simplified conceptual view of the fate of carbon in HWP is shown in Fig. 4.

The conceptual flow model started with harvested wood being taken out of the forest and undergoing manufacture and use by society and ended with carbon emission to the atmosphere. MitigAna tracks the fate of harvested carbon by its spatial origin and simulates carbon transfers in annual time steps.

Each element in Fig. 4 comprises a more complex structure (see Additional file 4) and some key components are described in detail below.

#### Utilization hierarchies

Utilization hierarchies have been developed to match industry practice and to improve the rationality of MitigAna. In Fig. 4 and Additional file 4: Figs. S3–S7, the hierarchy generally follows the rule that upper left is higher than lower right. HWPs in a higher position may be utilized the same way as the lower position but not vice versa.

MitigAna separates sawlogs and pulp logs into two distinct streams to explicitly indicate the utilization barrier between them. Pulp logs are smaller in diameter or have lower fiber quality, which is less suitable for sawnwood and plywood manufacturing. On the other hand, sawlogs may be used the same way as pulp logs, although it is normally not economically beneficial to do so. Milling residues and by-products are also explicitly represented in the model to enable more controls and constraints to be applied to the downstream products.

Further separating panel types into structural and non-structural categories recognizes the distinct raw material requirements for each product type. Structural panels generally are produced directly from logs. Plywood involves peeling the logs into veneers before drying, forming and pressing into panels. OSB requires directly stranding the log into particular length strands (e.g. 4–6″ long), which is a size requirement that chips, shavings and sawdust normally cannot achieve. In contrast, non-structural panels are often produced from wood chips and other by-products of primary wood processing.

#### Solid and composite wood end uses

Six solid and composite wood end-use categories were recognized in MitigAna (Additional file 4: Fig. S4). The resolution is constrained by data availability [10, 51, 52, 62, 109–114]. Table 3 provides examples of each end-use category.

**Table 3:** Examples of end-uses. CLT: cross laminated timber, Glulam: glue-laminated timber

End-use categories	Examples
Residential dwelling	Studs, joists, wall panels, staircase, cabinets
Residential renovation	Cabinets, doors, window frames, counter tops, floors
Non-residential building	CLT beams, GluLam beams, studs, I-beams
Furniture	Tables, chairs, shelves, nightstands, bed frames
Industrial production	Pallets, concrete forming, crates, barrels
Other end-uses	Electricity poles, fences, crossties

##### Residential renovation

Residential renovation is one of the major uses of wood products in the recent decade. Nearly 50% of the annual consumption of solid and composite wood products in Canada is for residential renovation [109, 114]. In the US, the largest importer of BC wood, renovation accounts for about 25% of their solid and composite wood products consumption [62].

Some models considered residential renovation as a separate pool with a specific carbon retention function parallel to the other end-uses (cf. [10, 93, 107, 115–119]). This approach implies that all the wood in the newly built dwellings would last until demolition and can cause double counting for at least one renovation cycle.

MitigAna subdivided the residential dwellings pool into *residential original* and *residential renovation* (Additional file 4: Fig. S4). *Residential original* referred to wood products that were in the dwelling since it was constructed and remained until the dwelling was demolished. *Residential renovation* referred to wood products that were subject to repair and remodeling over a dwelling’s service life. Although the partition introduces additional uncertainties, this approach provides a more conservative estimation of the dwelling carbon stock and is closer to the real-world carbon dynamics.

##### Carbon retention pattern for solid and composite wood end uses

It has been established that solid and composite wood end uses generally have an expected service life and the peak retirement rate happens near the expected service life instead of at the beginning [120, 121]. This carbon retention pattern is best described by the Gamma distribution with a shape parameter, “α”, and a scale parameter, “β”. The “α” and “β” values for residential dwellings in Canada were estimated to be 2.54 and 43.2 [121]. It has been noted that the estimated “α” values of the residential dwellings in the US, Canada and Norway do not vary greatly (2.07, 2.54 and 1.80, respectively) [121]. It was therefore conjectured that other solid and composite wood end uses may be described by Gamma distributions with similar shape parameters. When the “α” value is held constant, the one-parameter Gamma distribution is a subset of the natural exponential family, which is a common simplification practice of the distributions in the exponential family. Therefore, as a first approximation, the “α” values of all other end uses were assumed to be 2.54. The mean, “μ”, of the Gamma distribution (i.e. the mean service life), equals “α” multiplied by “β”. The mean service life was assumed to be equal to the expected service life. The “β” values were then calculated using μ divided by α (Table 4).

**Table 4:** Gamma parameters for various end uses

End uses	Expected Service Life (ESL)	μ	α	β	Reference for ESL
Residential original	110	110	2.54	43.2	[121]
Residential renovation	30	30	2.54	11.8	[109]
Non-residential building	75	75	2.54	29.5	[107]
Furniture	38	38	2.54	15	[107, 115]
Industrial production	3	3	2.54	1.18	[63]
Other end uses	25	25	2.54	9.84	[123]
US residential original	166	166	2.07	80.2	[121]
Japanese residential original	56	56	2.54	22	[140]

#### Pulp and paper

MitigAna distinguished the carbon flows in chemical and mechanical pulp production (Additional file 4: Fig. S5). With the same pulp output, the biogenic climate impact can vary twofold between chemical and mechanical processes due to burning black liquor in the recovery furnace.

##### Carbon retention for paper products

When applying the Gamma distribution to short-lived uses, the “α” value tends to be near “1”, which is close to the first order decay [120]. Consequently, the first order decay function was used for paper products. The half-life was assumed to be 2 years in line with previous publications and IPCC defaults [63, 107, 115, cf. 122, 123].

### Spatial and temporal tracking of carbon

MitigAna is used to track the fate of harvested carbon such that the emissions can be accounted and assigned to the area where the wood was harvested, and to record where (i.e. region or country) the HWP carbon is located in every time step. In this study, we simulated wood carbon dynamics among various jurisdictions through trade in annual time steps between 2016 and 2050. Both temporally static and dynamic data were used.

#### International trade

MitigAna is capable of handling either a production approach or a stock change approach.^8^ This research modeled the fate of carbon using the production approach to align with the current international reporting framework where BC is responsible for reporting emissions from exported HWPs regardless of where in the world these emissions occur [43, 82]. BC’s major wood trading partners modeled in MitigAna were the US, China, Japan and the EU. The other trading partners were combined as “other”.

In reality, any jurisdiction that imported BC wood can further export to another jurisdiction. From a modeling perspective, this activity is challenging to track and implement because, to our knowledge, there have been no data publicly available to distinguish multi-layer trading activities. Consequently, MitigAna only incorporated one layer of trading activity for this study.

##### China

The solid and composite wood products stream in China is significantly different from those of Canada, US and Japan. The MitigAna model adopted a jurisdiction-specific flow for the Chinese market (Additional file 4: Fig. S7) based on Manley and Evison [63].

The first order decay function was used for HWP in China [63, 123] and Table 5 summarizes the half-lives for each end use.

**Table 5:** End uses in China and their half-lives in years

End uses	Half-lives (years)
Temporary construction material, burnt after use	0.5
Temporary construction material with recycle	2.5
Packaging use	3
Appearance lumber	35
Appearance plywood, OSB, MDF and particleboard	25
Other short-lived uses	2

#### Study period

This study’s goal was to contrast the emission consequences of various *future* bioeconomy strategies. Historical HWPs storage and emissions arise from wood products have already been manufactured, traded and consumed. The *future* bioeconomy strategies investigated in this study are generally unable to influence the historical storage or emissions. The inherited emissions (i.e. those arising from wood harvested prior to the start of the analysis period) would be the same for all scenarios and therefore only the differences of future emissions are addressed here. 2016 was chosen as the base year. It was the latest year with data when this study was initiated. This year was considered as a suitable representation for BC’s forest products sector as the economy had recovered from the Global Financial Crisis, the wood products exports had not been significantly affected by the renegotiation of North American Free Trade Agreement (NAFTA) nor the escalated trade tensions with China. 2050 was chosen because it was the latest period for which most jurisdictions had defined their mitigation targets. Although the carbon storage benefits of long-lived uses of wood may have been revealed in more detail if the simulation was conducted over a longer time horizon, this study did not extend the study period to hundreds of years in order to focus on the next approximately 30 years.

#### Temporal data inputs and sources

The harvest rate projections were based on BC Ministry of Forests, Lands, Natural Resource Operations & Rural Development's (FLNRORD) compilations of annual allowable cut (AAC) projections and the fractions of realized AAC available in 2016. The scope of this study was limited to the production, trade and usage decisions along the post-harvest wood products supply chain. It did not explore alternative forest management and timber harvest strategies. The harvest projections were held constant among the scenarios analyzed. This study also did not explore alternative uses of bark other than hog fuel. Although pre-treatment is possible [125], published literature has indicated that biofuel conversion requires clean fiber feedstock with low bark content and soil contamination [78, 126]. The utilization of bark was not considered a priority in this study [127–129].

A volumetric flow analysis was conducted with the goal of capturing the partitioning information in percentage form for the first two categories (see Additional file 1). The analysis was conducted using the data compiled from BC mill surveys [11]. The recovery factors were acquired from LCA studies [14, 15, 130–132], and forest products conversion factors [12], in addition to the mill surveys.

The end-use partitions were compiled from several Canadian Wood Council (CWC) and US Department of Agriculture (USDA) joint-research reports [10, 51, 52, 62, 109–113] and the Forest Economic Advisors (FEA) [114].

Population data were collected from Statistics Canada (StatCan) [34]. The market share of timber constructions was compiled from Elling et al., McKeever et al. and Canadian Institute of Steel Construction (CISC) market share report [50–52]. International trade data for BC’s HWPs were collected from StatCan [133], Forestry Innovation Investment (FII) [44] and FPInnovations [49]. Different market growth and policy implications were examined and these resulted in dynamic carbon allocations being applied to assess the impacts. Technological advancement in wood products processing was not the focus of this study, and while future improvements in conversion efficiencies are likely, these were not included here.

This study did not explore alternative end-of-life practices and assumed all retired wood materials were sent to landfills. In addition, landfilled woody biomass was treated as instantaneous emissions, consistent with UNFCCC adopted decisions for the Kyoto Protocol [43] and the way in which Canada’s GHG Inventory is reported [93]. Uncertainty analysis has indicated that landfill parameters have a significant impact on emission estimations, but the amount of postconsumer HWPs that were sent to landfills, the decomposable fraction of degradable organic carbon (DOC_f_), and the amount of methane leakage remain highly uncertain [115].

Several studies have modelled the landfill dynamics in Canada (e.g. [30, 105, 107, 118]). They suggested that over 60% of the postconsumer HWPs were sent to landfills [10, 30]. However, some jurisdictions have been actively discouraging wood in landfills and encouraging energy recovery [134–136]. The default DOC_f_ value previously recommended by the IPCC was 50% [123], but recent studies have suggested much lower values (less than 10%) for wood [19, 115, 137, 138]. Engineered landfills have methane capture technology that can substantially reduce methane emissions because the global warming potential for CH_4_ over 100 years is 28 times higher than CO_2_. However, not all landfills are engineered and the efficacy of the methane capture nation-wide or globally is unknown. A ~ 4% leakage of methane will offset the rest of the landfill carbon storage benefit.

We decided to account for landfilled HWPs on the basis of instantaneous oxidation in this paper and investigate it in future work so that (1) the emission results are comparable to previous reported Canadian values, (2) the emissions increase/decrease of each scenario relative to the baseline are “traceable” to the changes of utilization or trade behavior outlined in the bioeconomy scenarios, and (3) the effects of landfills can be independently identified when future analyses are conducted.

### Biomass supply reallocation rules for the inward-focused scenarios

In order to meet the domestic demand established in the inward-focused scenarios, some of the exported HWPs needed to be shifted to domestic uses. This reallocation commenced with roundwood as increasing the consumption of logs domestically was expected to provide additional carbon benefits. Additional processing of wood products generally adds value to the product and reallocating logs to domestic use would likely create additional jobs and economic value for the province [139].

If all exported logs were reallocated to domestic manufacturing but still could not fulfill the demand of a particular product established in the scenario, the export of this products was reallocated to meet the domestic demand.

If a complete shift of the exported amount still could not adequately meet the domestic demand, then the biomass sent to the competing products^9^ was reallocated to produce this specific product, provided that some criteria were met:

- the competing product had a shorter service life, and/or
- the competing product had a smaller substitution effect.

## Supplementary Information

**Additional file 1.** Flow analysis.

**Additional file 2.** Brock Commons calculation.

**Additional file 3.** Renewable transportation fuel calculation.

**Additional file 4.** MitigAna structure and PyDS.

## Data Availability

The datasets used in this study are publically available and cited in “Methods” section.

## Abbreviations

ALL_CONS: Scenario label: all forest biomass manufactured to construction materials
ALL_FUEL: Scenario label: all forest biomass as feedstock for renewable fuel
BASE: Scenario label: the business-as-usual baseline
BC: The province of British Columbia in Canada
CBMF-HWP: Carbon Budget Modeling Framework for Harvested Wood Products
CLT: Cross laminated timber
DOC_f_: Fraction of degradable organic carbon which decomposes
EU: European Union
GHG: Greenhouse gas
GluLam: Glue laminated timber
HTL: Hydrothermal liquefaction
HWPs: Harvested wood products
IN_CONS: Scenario label: inward-focused, increase domestic market share of wood-frame construction
IN_FUEL: Scenario label: inward-focused, prioritize renewable fuel feedstock
IN_PCF: Scenario label: inward-focused, population-driven, construction-dominated biofuel-subordinated
IN_POP: Scenario label: inward-focused, demand increase driven by population growth
LCA: Life cycle assessment
LVL: Laminated veneer lumber
MDF: Medium density fibreboard
Mt: Mega tonnes
MtCO_2_e: Mega tonnes of carbon dioxide equivalent
OSB: Oriented strand board
OSL: Oriented strand lumber
OU_CN: Scenario label: outward-focused, prioritize export to China
OU_CONS: Scenario label: outward-focused, prioritize export to US and other markers for constructions
OU_PLTS: Scenario label: outward-focused, prioritize wood pellets export to EU and Japan for energy
yr: Year

## Footnotes

1 The quantitative estimates of carbon retention times in various HWPs are described in “Methods” section.

2 In contrast to international market, domestic market means the market of BC and other regions in Canada. To restrict the biomass origin to BC and avoid ambiguity, domestic demand or domestic consumption in this paper refers to wood products that are harvested in BC, and consumed in BC and elsewhere in Canada.

3 see “Methods” section for estimate of carbon retention time in various HWPs.

4 and its subsequent mills such as cross-laminated timber (CLT) plant.

5 Brock Commons Tallwood House is an 18-story hybrid mass timber construction in BC. The estimation is based on wood volume. Brock Commons used 2233 m^3^ of CLT and Glulam [84, 85]. This scenario sends annually roughly 21 million m^3^ yr^−1^ of sawnwood to construction sites. Detailed calculations are presented in Additional file 2.

6 The energy demand of BC’s transportation sector is 334.45 PJ and Canada’s is 2601.13 PJ [91]. The energy content of renewable fuel is 35.6 MJ L^−1^ and the fuel yield is roughly 12.5 kJ (g forest biomass)^−1^ [78, 80, 92]. Detailed calculations are presented in Additional file 3.

7 This point can be visualized in Fig. 3 by comparing the red bars in the BASE scenario (or IN_POP, IN_CONS, OU_CN) to the OU_CONS scenario (or OU_PLTS), because the OU_CONS and OU_PLTS scenarios assumed that only the domestic demand for pulp and paper were fulfilled.

8 For the accounting approaches, see Pingoud et al. [124] and IPCC [123].

9 Competing products are HWPs that are manufactured from the same raw material. One product therefore competes for the biomass supply with the other. For example, MDF mills and pulp mills may compete for fibre supply from pulp logs.
