# Supplementary material for: Inward- versus outward-focused bioeconomy strategies for British Columbia’s forest products industry: a harvested wood products carbon storage and emission perspective
Source: Carbon Balance Manag. 2021 Sep 25;16:30. doi: 10.1186/s13021-021-00193-4 (PMC8466961; doi:10.1186/s13021-021-00193-4)
Supplement: Supplementary file 1 — Additional file 1. Flow analysis. [file 13021_2021_193_MOESM1_ESM.pdf]

Additional File 1 Flow Analysis

Fig. S1 is a volumetric flow analysis of BC's harvested wood products supply chain up to the commodity level. The unit in Fig. S1 is thousand cubic meters roundwood equivalent under bark (000 m^3 rwe u.b.). The flow starts from the left upper corner with 67,826,000 m^3 rwe u.b. harvested from BC's forest in 2016. The green color indicates that the physical state of the biomass is roundwood. Roundwood were consumed to produce sawnwood, plywood, OSB, pulp and by-products. The light blue column indicates the primary product outputs from various types of mills. The export of these primary products are not shown in this table. The light orange column indicates the wood chip outputs from mills. Wood chips (23,934,000 m^3 rwe u.b.) were allocated to pulp and paper mills. The yellow column indicates the sawdust outputs from mills. Sawdust was allocated to pulp and paper mill, MDF mills and wood pellet mills. Sawdust may be consumed onsite of the mills as bioenergy, however high resolution mill-specific data were not always available (wood fuel use in MDF and OSB mills were estimated from LCA studies). Fig. S1 shows the bioenergy use of sawdust as an individual category. Energy uses were collected in the orange column. Light green and blue column indicate the production volume of chemical (10,111,000 m^3 rwe u.b.) and mechanical pulp (1,250,000 m^3 rwe u.b.). Only a fraction of the pulp produced were consumed for paper manufacturing in BC (2,691,000 m^3 rwe u.b. and 813,000 m^3 rwe u.b.), and the rest was exported.

Fig. S2 is a Sankey diagram of carbon flow of BC's harvested wood products supply chain in 2016. It is plotted using the output data of the MitigAna model.

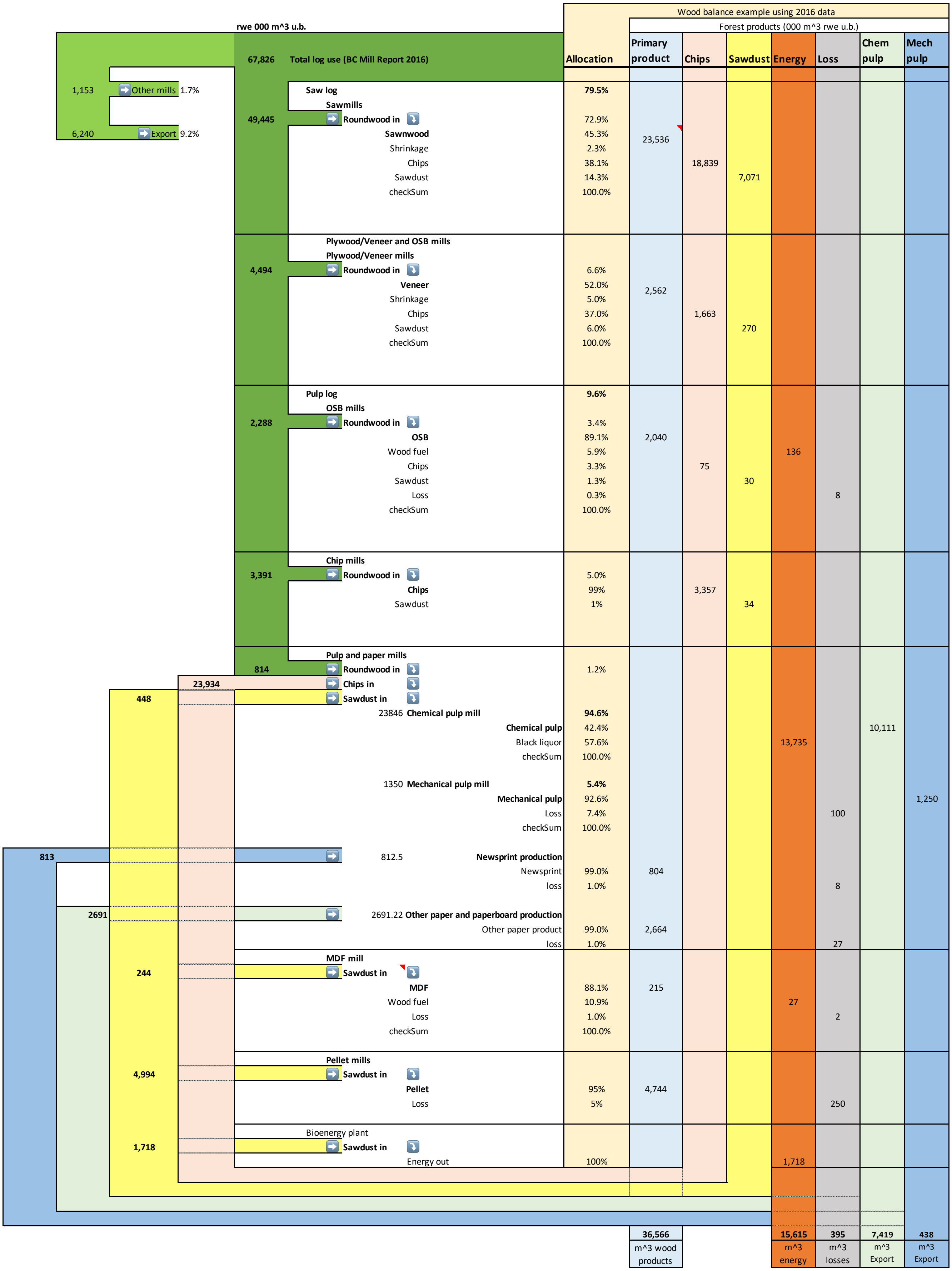

Figure S1. BC woody biomass flow, production and yield.

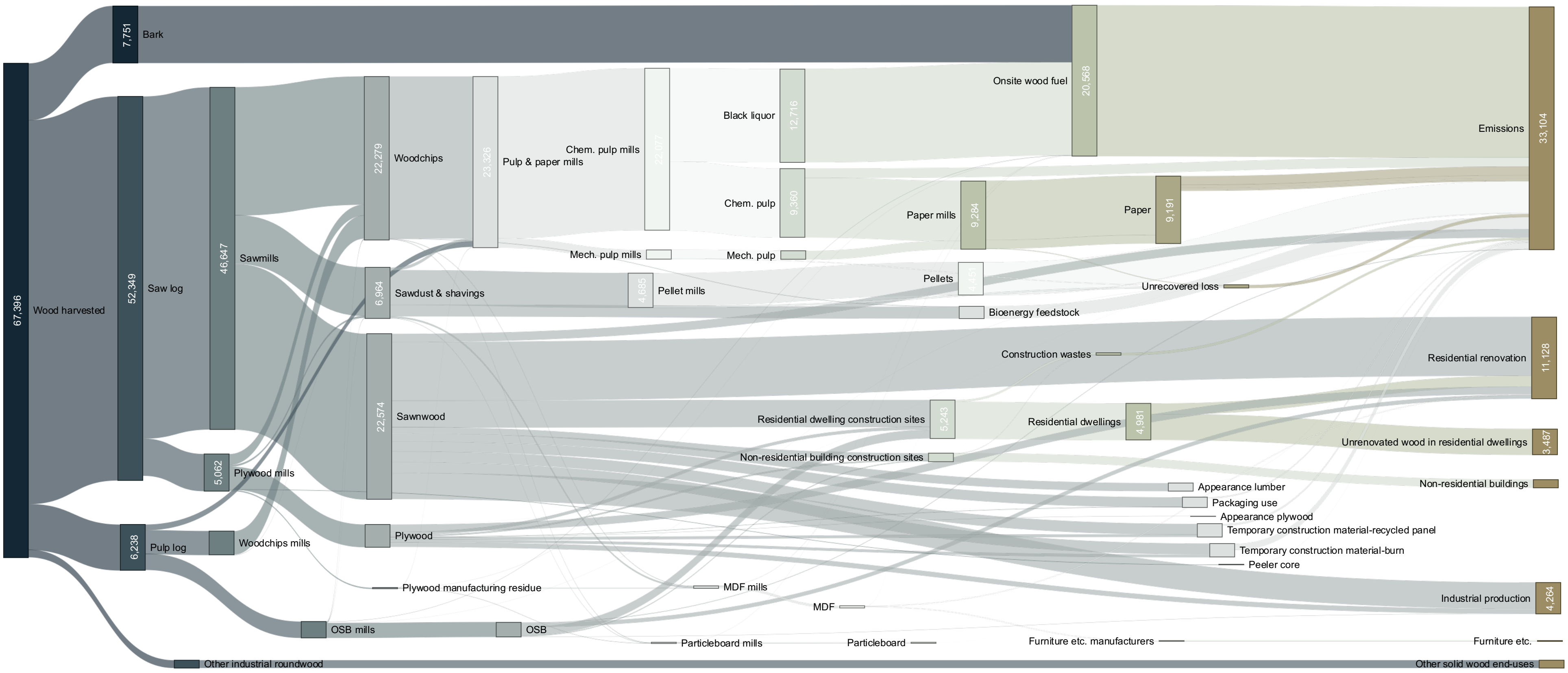

Figure S2. A Sankey diagram of MitigAna output of 2016 flux in supply chain without geo locations. Unit: $ktCO_2e$ year^{-1}.
