# Supplementary material for: Inward- versus outward-focused bioeconomy strategies for British Columbia’s forest products industry: a harvested wood products carbon storage and emission perspective
Source: Carbon Balance Manag. 2021 Sep 25;16:30. doi: 10.1186/s13021-021-00193-4 (PMC8466961; doi:10.1186/s13021-021-00193-4)
Supplement: Supplementary file 2 — Additional file 2. Brock Commons calculation. [file 13021_2021_193_MOESM2_ESM.pdf]

Additional File 2 Brock Commons Calculation

Description	Value	Unit	Reference
Carbon content of wood	0.5	tC (t wood)$^{-1}$	[1]
CLT and Glulam (Douglas fir) density	0.470	t m^{-3}	[1]
Volume of wood products used in Brock Commons (CLT and GluLam combined)	2233	m^3	[2]
Average annual sawnwood products sent to residential dwellings in the ALL_CONS scenario	5,340,841	tC year^{-1}	This study
	10,681,682	t wood year^{-1}	
	22,726,984	m^3 year^{-1}	
Average annual number of residential buildings required to be built under the ALL_CONS scenario relative to Brock Commons (bce) in terms of structural wood consumption	10,178	bce year^{-1}	
Average annual sawnwood products sent to residential dwellings in the OU_CONS scenario	4,865,773	tC year^{-1}	This study
	9,731,546	t wood year^{-1}	
	20,705,417	m^3 year^{-1}	
Average annual number of residential buildings required to be built under the OU_CONS scenario relative to Brock Commons (bce) in terms of structural wood consumption	9,272	bce year^{-1}	

bce: Brock Commons equivalent.

Reference

1. Bowick M. Brock Commons Tallwood House, University of British Columbia: An environmental building declaration according to EN 15978 standard [Internet]. Athena Sustainable Materials Institute; 2018 Jan. Available from: http://www.athenasmi.org/news-item/environmental-building-declarations-for-ubc/

2. FII. Brock Commons Tallwood House: Factsheets [Internet]. Forestry Innovation Investment; 2017 [cited 2018 Mar 13]. Available from: https://www.naturallywood.com/sites/default/files/documents/resources/brock_commons_tallwood_house_aug_2017-web.pdf
