# Supplementary material for: Inward- versus outward-focused bioeconomy strategies for British Columbia’s forest products industry: a harvested wood products carbon storage and emission perspective
Source: Carbon Balance Manag. 2021 Sep 25;16:30. doi: 10.1186/s13021-021-00193-4 (PMC8466961; doi:10.1186/s13021-021-00193-4)
Supplement: Supplementary file 3 — Additional file 3. Renewable transportation fuel calculation. [file 13021_2021_193_MOESM3_ESM.pdf]

ADDITIONAL FILE 3 RENEWABLE TRANSPORTATION FUEL CALCULATION

Renewable Fuel Yield Calculation - Pyrolysis Pathway

Pyrolysis Pathway	Parameter name	Value	Unit	Reference	Notes
Data from the literature	Pyrolysis plant yield	1.65	kg forest residue input (L bio-oil output)$^{-1}$	[1]	bio-oil is the intermediate product that needs to be upgraded to the final fuel
	Bio-oil density	1200	g L^{-1}	[2–4]	
	Upgrading plant yield	2.14	kg bio-oil input (L final fuel output)$^{-1}$	[1]	
	S-P-F Density	378	kg m^{-3}	[5]	
Calculations	Pyrolysis plant yield unit conversion	1.38	kg forest residue input (kg bio-oil output)$^{-1}$		Pyrolysis plant yield / (bio-oil density / 1000), bio-oil not final fuel
	Overall yield	2.94	kg forest residue input (L final fuel output)$^{-1}$		Pyrolysis plant yield unit conversion * upgrading plant yield
	Overall yield conversion	128.46	L final fuel output (m^{-3} wood input)		S-P-F Density / Overall yield

Renewable Fuel Yield Calculation - Hydrothermal Liquefaction (HTL) Pathway

HTL Pathway	Parameter name	Value	Unit	Reference	Notes
Data from the literature	Forest residue to bio-oil	0.37	kg bio-oil output (kg forest residue input)$^{-1}$	[3]	Converting from forest residue to intermediate bio-oil
	Forest residue to wood pellet	0.89	kg wood pellet output (kg forest residue input)$^{-1}$	[6]	Converting from forest residue to wood pellet
	Wood pellet to bio-oil	0.37	kg bio-oil output (kg wood pellet input)$^{-1}$	[3]	Converting from wood pellet to intermediate bio-oil
	Bio-oil to final oil	0.75	weight%	[3]	
	S-P-F Density	378	kg m^{-3}	[5]	
	Final fuel density	826.00	kg m^{-3}	[7]	This is a blended value of renewable gasoline, jet fuel, Diesel and by-product heavy oil
Calculations	Forest residue to final fuel yield	0.28	kg final fuel output (kg forest residue input)$^{-1}$		Not convert to wood pellet first for transportation
	Forest residue to wood pellet, then final fuel yield	0.24	kg final fuel output (kg forest residue input)$^{-1}$		Convert to wood pellet first for transportation
	Forest residue to final fuel yield conversion	125.96	L final fuel output (m^{-3} wood input)		Not convert to wood pellet first for transportation
	Forest residue to wood pellet, then final fuel yield conversion	112.11	L final fuel output (m^{-3} wood input)		Convert to wood pellet first for transportation

Renewable fuel meeting energy demand of BC's transportation sector

	Parameter name	Value	Unit	Value	Unit	Reference	Notes
Data available	HHV of renewable fuel	35.57	MJ L^{-1}			[2–4]	
	Yield	122.18	L final fuel output (m^{-3} of wood input)				Average of pyrolysis and HTL yield
	Bioenergy feedstock in IN_FUEL scenario	27,479,862	tCO_2e $year^{-1}$				Taken from inward-focused, renewable fuel scenario. All pulp log and milling residue are sent to bioenergy feedstock, all time average
	Bioenergy feedstock in ALL_FUEL scenario	53,827,483	tCO_2e $year^{-1}$				Taken from all harvest to renewable fuel scenario. All the harvest is sent to bioenergy feedstock, all time average
	Bioenergy feedstock in IN_PCF scenario	25,301,614	tCO_2e $year^{-1}$				Taken from inward construction-dominated biofuel subordinated scenario, all time average
	BC's transportation demand	334.45	PJ			[8]	
	S-P-F Density	378.00	kg m^{-3}			[5]	
	Wood carbon content	0.51				[2–4]	
Calculations	Yield conversion	6.15	MJ final fuel output (kg				

			CO_2e of wood input)$^{-1}$				
	Renewable fuel output of IN_FUEL scenario	168.95	PJ year^{-1}	4749.74	Million L year^{-1}		
	Renewable fuel output of ALL_FUEL scenario	330.94	PJ year^{-1}	9303.77	Million L year^{-1}		
	Renewable fuel output of IN_PCF scenario	155.56	PJ year^{-1}	4373.24	Million L year^{-1}		
	BC coverage of IN_FUEL scenario	51%					
	BC coverage of ALL_FUEL scenario	99%					
	BC coverage of IN_PCF scenario	47%					

Reference

1. Ringsred A. The potential of the aviation sector to reduce greenhouse gas emissions by using biojet fuels [Internet] [MASc Thesis]. [Vancouver BC]: University of British Columbia; 2018 [cited 2018 Jul 18]. Available from: https://open.library.ubc.ca/collections/ubctheses/24/items/1.0368545

2. Cox K, Renouf M, Dargan A, Turner C, Klein-Marcuschamer D. Environmental life cycle assessment (LCA) of aviation biofuel from microalgae, Pongamia pinnata, and sugarcane molasses. Biofuels, Bioproducts and Biorefining [Internet]. 2014 [cited 2018 Aug 1];8:579–93. Available from: http://doi.wiley.com/10.1002/bbb.1488

3. Tews IJ, Zhu Y, Drennan C, Elliott DC, Snowden-Swan LJ, Onarheim K, et al. Biomass Direct Liquefaction Options. TechnoEconomic and Life Cycle Assessment [Internet]. 2014 Jul. Report No.: PNNL-23579, 1184983. Available from: http://www.osti.gov/servlets/purl/1184983/

4. BC Hydro. Site C Clean Energy Project [Internet]. [cited 2019 Jul 26]. Available from: https://www.sitecproject.com/document-library/information-materials

5. Nielson RW, Dobie J, Wright DM. Conversion Factors for the Forest Products Industry in Western Canada. Vancouver, BC: Forintek Canada Corp.; 1985 Mar.

6. Pa AA. Development of British Columbia wood pellet life cycle inventory and its utilization in the evaluation of domestic pellet applications [Internet] [MASc Thesis]. [Vancouver BC]: University of British Columbia; 2010 [cited 2019 Jul 26]. Available from: https://open.library.ubc.ca/collections/ubctheses/24/items/1.0058986

7. Nie Y, Bi X. Life-cycle assessment of transportation biofuels from hydrothermal liquefaction of forest residues in British Columbia. Biotechnology for Biofuels [Internet]. 2018 [cited 2018 Apr 26];11. Available from: https://biotechnologyforbiofuels.biomedcentral.com/articles/10.1186/s13068-018-1019-x

8. NEB. Canada's Energy Future 2018: Energy Supply and Demand Projections to 2040 End - Use Demand [Internet]. National Energy Board. 2018 [cited 2018 Oct 31]. Available from: https://apps.neb-one.gc.ca/ftrppndc/dflt.aspx?GoCTemplateCulture=en-CA
