# Supplementary material for: Inward- versus outward-focused bioeconomy strategies for British Columbia’s forest products industry: a harvested wood products carbon storage and emission perspective
Source: Carbon Balance Manag. 2021 Sep 25;16:30. doi: 10.1186/s13021-021-00193-4 (PMC8466961; doi:10.1186/s13021-021-00193-4)
Supplement: Supplementary file 4 — Additional file 4. MitigAna structure and PyDS. [file 13021_2021_193_MOESM4_ESM.pdf]

ADDITIONAL FILE 4 MITIGANA STRUCTURE AND PYDS

SOLID AND COMPOSITE WOOD COMMODITIES

A partial view of the carbon flow focusing on the solid and composite wood commodity stream is shown in Fig. S3.

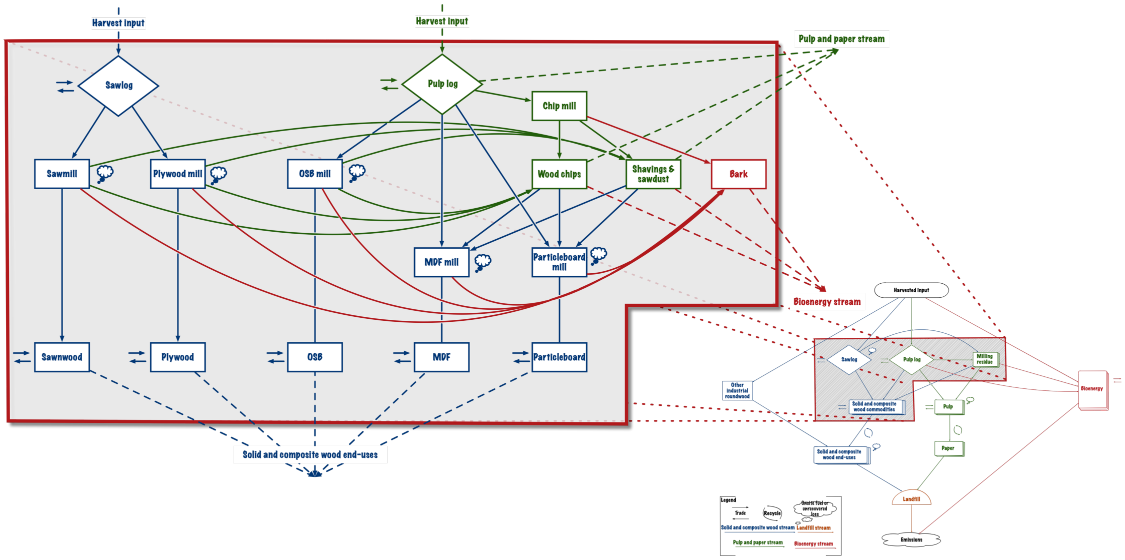

Figure S3. Carbon flow of the solid and composite wood products commodities stream.

The taxonomy of harvested wood products published by Cohen and Ellis [1, 2] was generally followed with reference to FAO's forest products definitions [3]. However, the taxonomy was compromised by the low resolution of some reported production and consumption data with some of the product categories having to be combined. In particular, sawnwood included solid and glued lumber products such as lumber, timber, boards, finger joined lumber and edge glued panels, and engineered lumber-timber products such as glue-laminated timber (Glulam) and Cross-laminated timber (CLT). Plywood was used to refer to all the veneer-based products which also included laminated veneer lumber (LVL). Similarly, OSB referred to all the strand-based products, including oriented strand lumber (OSL). These aggregated commodity categories can be disaggregated in the future if data become available.

SOLID AND COMPOSITE WOOD END-USES

Fig. S4 illustrates the carbon flow in the solid and composite wood products end-use categories.

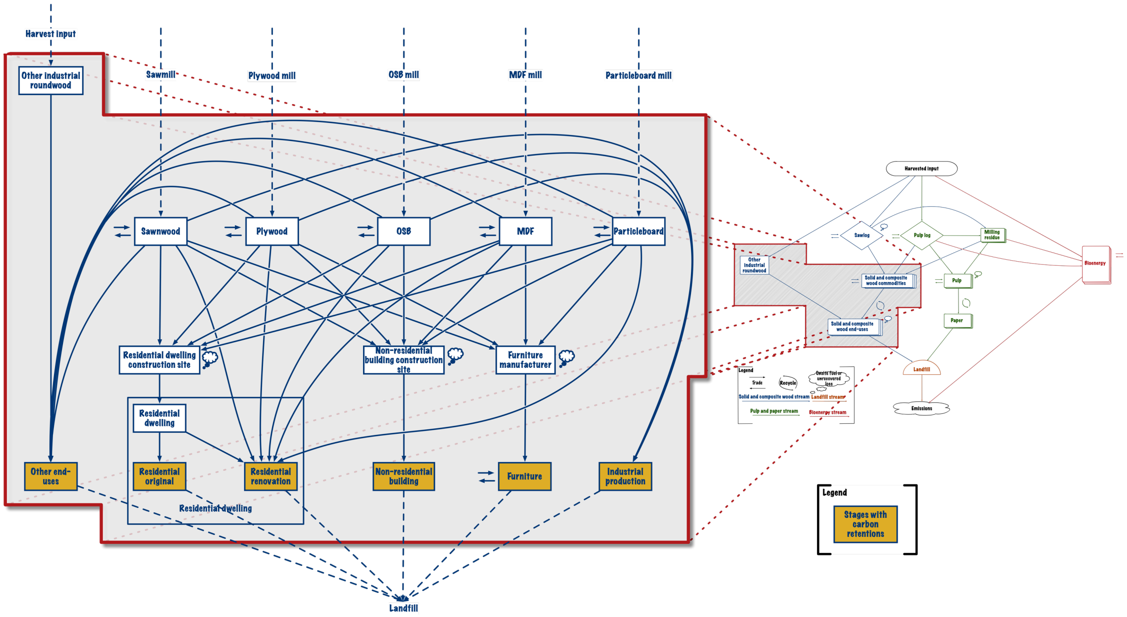

Figure S4. Carbon flow of the solid and composite wood products end-uses.

PULP AND PAPER

Fig. S5 describes the carbon flow in the pulp and paper stream.

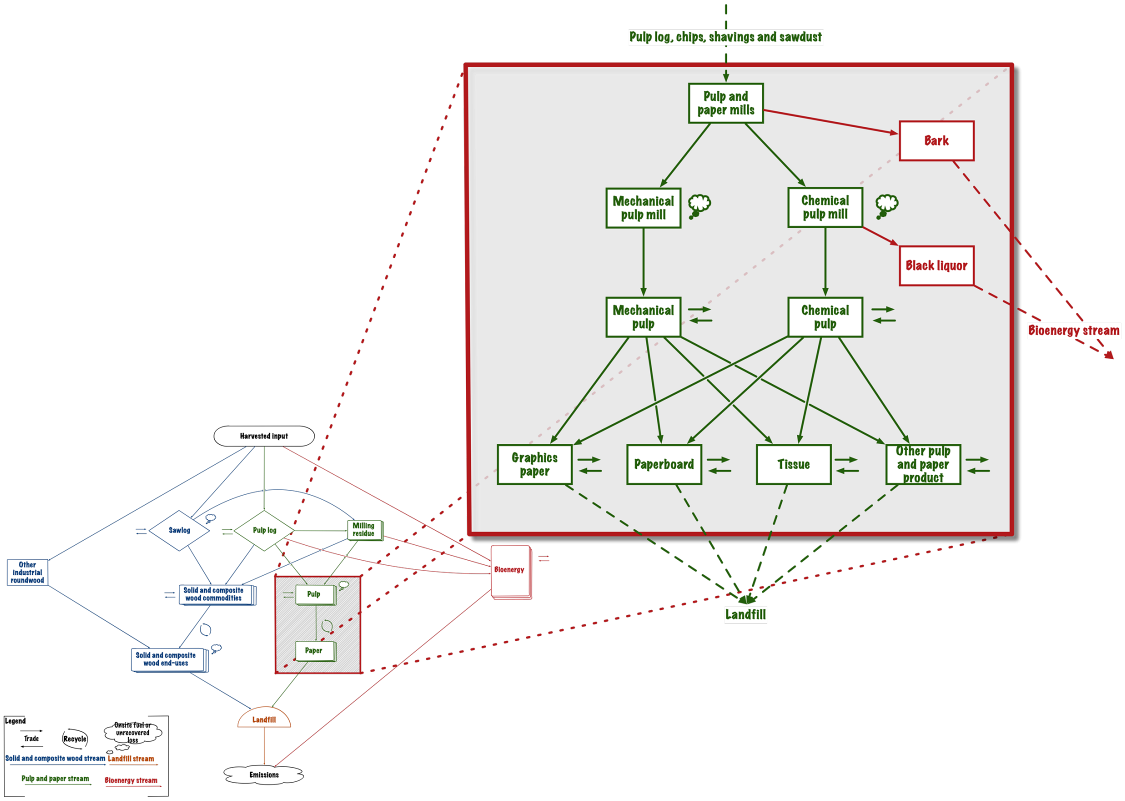

Figure S5. Carbon flow of the pulp and paper stream.

BIOENERGY

Fig. S6 demonstrates the carbon flow of wood-based bioenergy products. Bioenergy was assumed to be instantaneously oxidized (i.e. within the year of harvest).

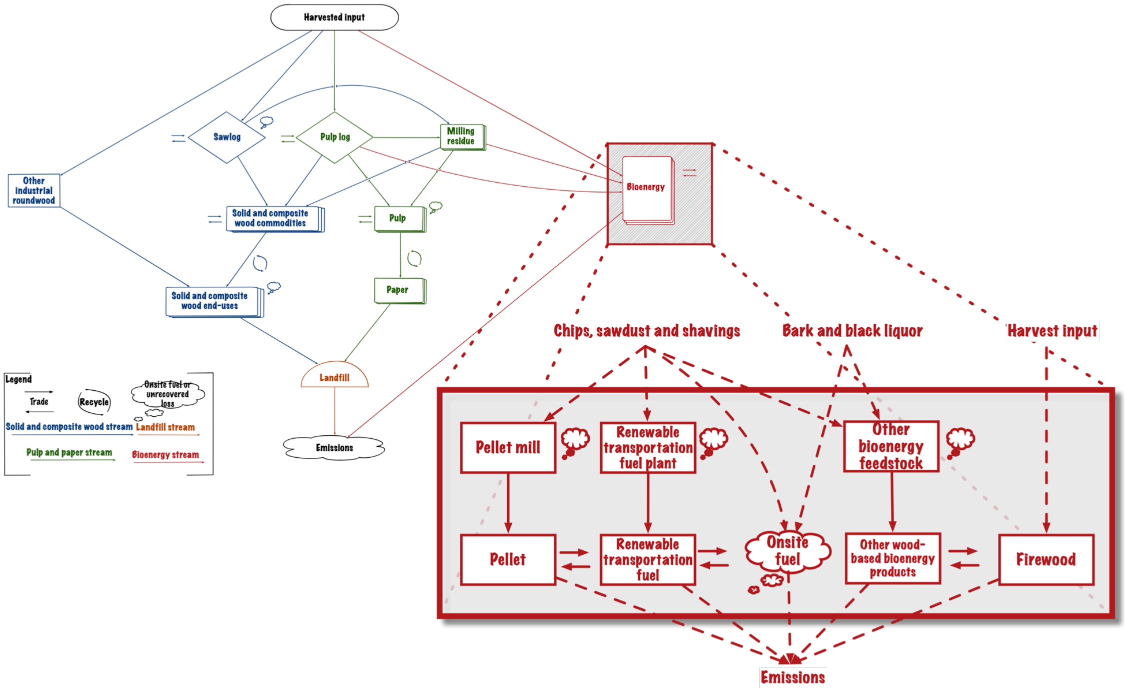

Figure S6. Carbon flow of the bioenergy stream.

CHINA

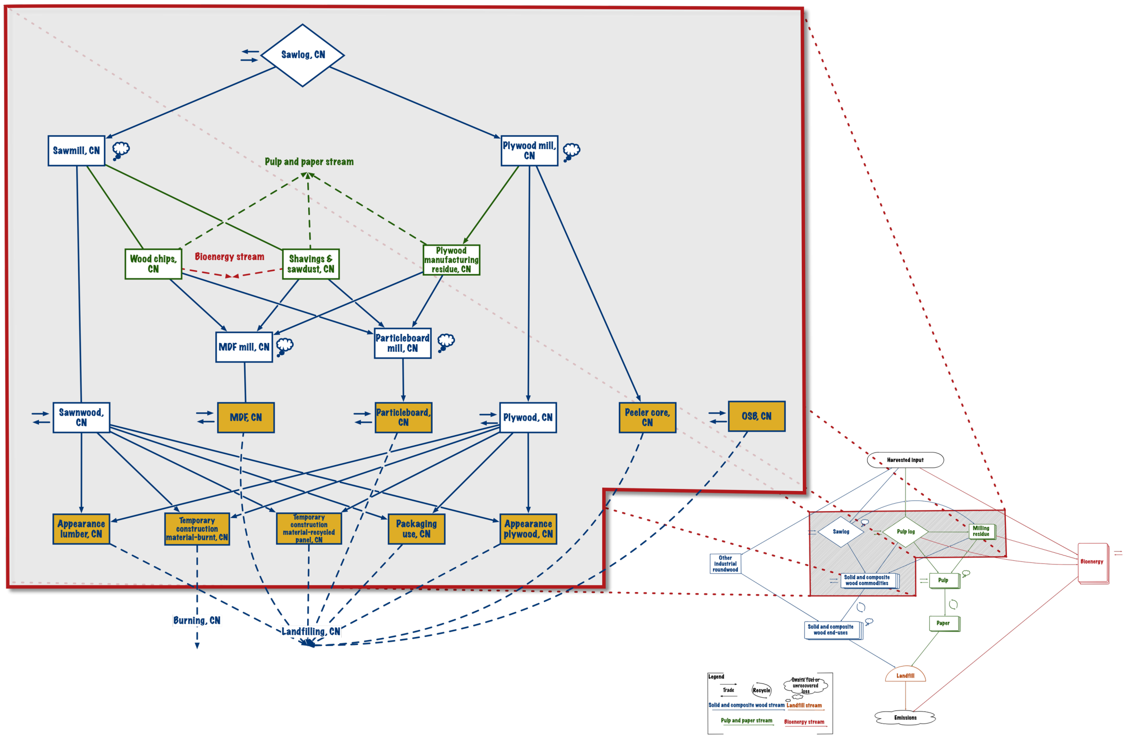

Figure S7. Carbon flow of the solid and composite wood products stream in China.

The "temporary construction material" application is similar to the category of "industrial production" in Fig. S4. These temporary construction material uses are not structural or non-structural wood applications in wood constructions. Examples are scaffolding, concrete forming and pallets. They are burnt or recycled at the end of their life.

SIMULATION STEPS

The workflow of the model runs for this study are described in Fig. S8.

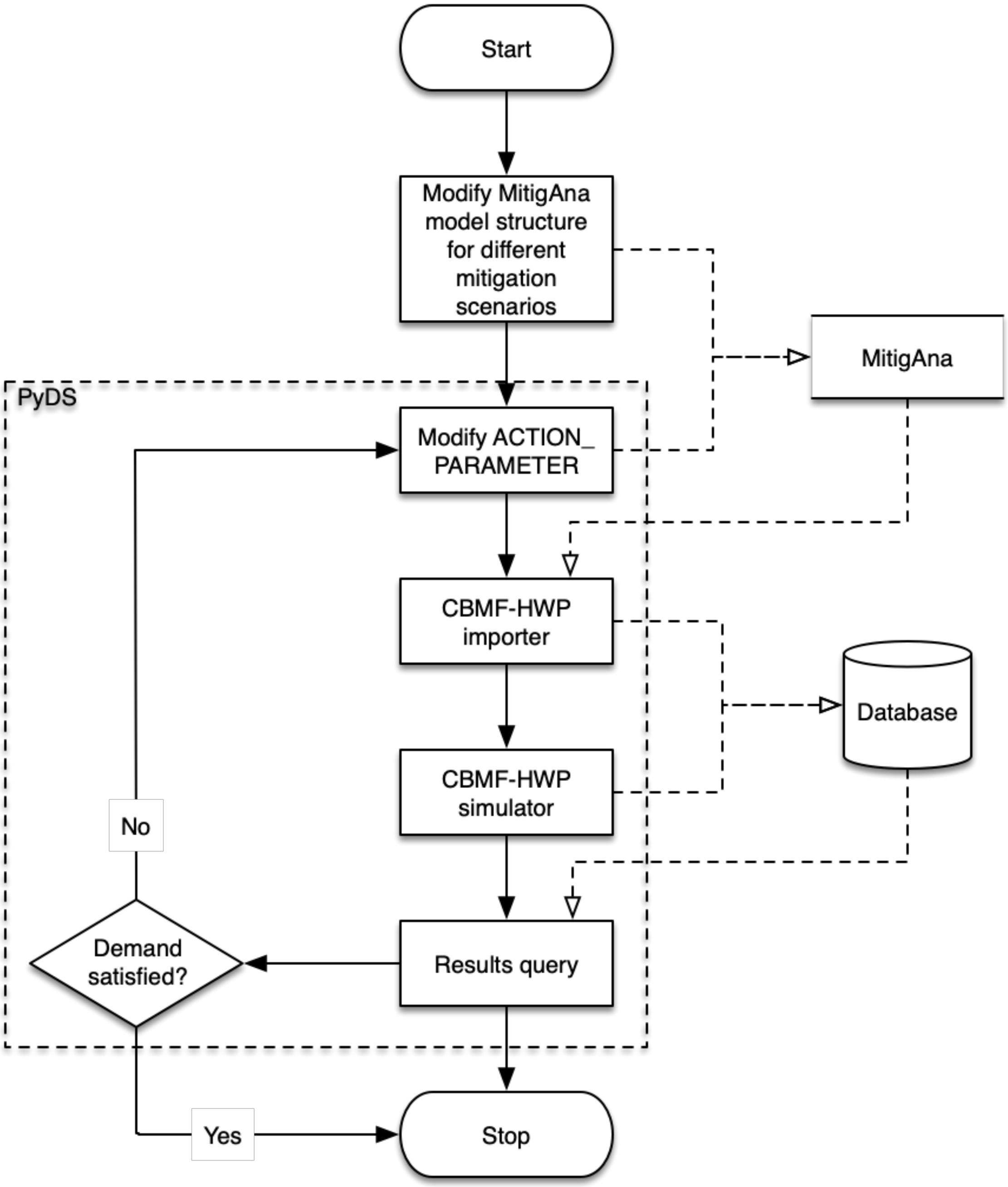

Figure S8. Flowchart of the mitigation model runs (PyDS: python demand search tool; CBMF-HWP: carbon budget modeling framework for harvested wood products; MitigAna: a harvested wood products carbon dynamics model for mitigation analysis).

The process began with modifying the MitigAna model structure for different requirements established in the mitigation scenarios. The action parameter time series were modified to satisfy different HWP's demands in different scenarios. Output of the CBMF-HWP simulator was queried to determine whether the domestic demand was satisfied or not. The biomass reallocation decisions made in this study were conducted using a top-down design with a binary search algorithm, implemented in Python (i.e. PyDS). If

demand was not satisfied, the PyDS module would initiate a reallocation process and repeat the simulation until the demand was satisfied. The scenario results were then compiled.

REFERENCES

1. Cohen DH, Ellis SC. A Revised Taxonomy of Wood Products 2008 edition [Internet]. Vancouver BC Canada: University of British Columbia, Natural Resources Canada, BC Ministry of Forests; 2008 Aug p. 77. Available from: https://cfs.nrcan.gc.ca/publications?id=4351

2. NRCan. Taxonomy of wood products [Internet]. Natural Resources Canada. Natural Resources Canada; 2014 [cited 2020 Aug 18]. Available from: https://www.nrcan.gc.ca/our-natural-resources/forests-forestry/forest-industry-trade/forest-products-applications/taxonomy-wood-products/14510

3. FAO. FAOSTAT Forest Products Definitions [Internet]. Food and Agriculture Organization of the United Nations; 2015. Available from: http://www.fao.org/forestry/statistics/80572/en/
